# Oncopig bladder cancer cells recapitulate human bladder cancer treatment responses *in vitro*

**DOI:** 10.3389/fonc.2024.1323422

**Published:** 2024-02-26

**Authors:** Natália V. Segatto, Lucas D. Simões, Camila B. Bender, Fernanda S. Sousa, Thais L. Oliveira, Júlia D. F. Paschoal, Bruna S. Pacheco, Isadora Lopes, Fabiana K. Seixas, Aisha Qazi, Faith M. Thomas, Sulalita Chaki, Noah Robertson, Jordan Newsom, Shovik Patel, Laurie A. Rund, Luke R. Jordan, Courtni Bolt, Kyle M. Schachtschneider, Lawrence B. Schook, Tiago V. Collares

**Affiliations:** ^1^ Technology Development Center, Laboratory of Cancer Biotechnology, Federal University of Pelotas, Pelotas, Rio Grande do Sul, Brazil; ^2^ Department of Animal Sciences, University of Illinois, Urbana, IL, United States; ^3^ Albion College, Albion, MI, United States; ^4^ Sus Clinicals Inc., Chicago, IL, United States

**Keywords:** Oncopig cancer model, bladder cancer, cisplatin, doxorubicin, gemcitabine, microarray, *in silico*

## Abstract

**Introduction:**

Bladder cancer is a common neoplasia of the urinary tract that holds the highest cost of lifelong treatment per patient, highlighting the need for a continuous search for new therapies for the disease. Current bladder cancer models are either imperfect in their ability to translate results to clinical practice (mouse models), or rare and not inducible (canine models). Swine models are an attractive alternative to model the disease due to their similarities with humans on several levels. The Oncopig Cancer Model has been shown to develop tumors that closely resemble human tumors. However, urothelial carcinoma has not yet been studied in this platform.

**Methods:**

We aimed to develop novel Oncopig bladder cancer cell line (BCCL) and investigate whether these urothelial swine cells mimic human bladder cancer cell line (5637 and T24) treatment-responses to cisplatin, doxorubicin, and gemcitabine in vitro.

**Results:**

Results demonstrated consistent treatment responses between Oncopig and human cells in most concentrations tested (p>0.05). Overall, Oncopig cells were more predictive of T24 than 5637 cell therapeutic responses. Microarray analysis also demonstrated similar alterations in expression of apoptotic (GADD45B and TP53INP1) and cytoskeleton-related genes (ZMYM6 and RND1) following gemcitabine exposure between 5637 (human) and Oncopig BCCL cells, indicating apoptosis may be triggered through similar signaling pathways. Molecular docking results indicated that swine and humans had similar Dg values between the chemotherapeutics and their target proteins.

**Discussion:**

Taken together, these results suggest the Oncopig could be an attractive animal to model urothelial carcinoma due to similarities in in vitro therapeutic responses compared to human cells.

## 1 Introduction

Bladder cancer is the most common neoplasm of the urinary tract and the tenth most common tumor in the population worldwide (1). Although patients with urothelial carcinoma have a wide range of therapeutic alternatives, such as surgery, chemotherapies and immunotherapy (2–4), acquired resistance and recurring adverse effects make long term treatment difficult, potentially leading to more serious adverse effects (5, 6). In addition, the need for lifelong surveillance, high rates of disease recurrence and resistance to chemotherapy make bladder cancer one of the malignancies with the highest cost of lifelong treatment per patient (7). Therefore, new, effective, selective, and economic therapeutic approaches for this neoplasia are needed.

In this context, biological models are an essential step in the research and development process of new potential therapies. The ability of biological models to mimic the human cellular response to therapies is critical for suitable disease models. Currently, the most widely used animal models in bladder cancer research are murine models, due to their small size, known genetics, ease of handling and low cost (8). Such models can be developed using several methods (9). However, it is necessary to recognize that mouse models are imperfect in their ability to translate results to clinical practice. For example, there are numerous discrepancies between humans and rodents, including drug metabolism rates (10), size, and cancer genetics (11, 12). In addition to murine models, canine models of spontaneous invasive muscle urothelial carcinoma deserve attention as well. Among their advantages, we highlight canine’s urothelial carcinoma similarities towards human muscle invasive urothelial carcinomas as it relates to clinical symptoms, cellular and pathological characteristics, response to chemotherapy and even shared molecular targets (13). However, canine spontaneous urothelial carcinomas are considered rare since they represent only 2% of all canine cancers (14). Hence, there is an urgent need to develop new robust and inducible urothelial carcinoma models.

Pigs represent an ideal animal model for the development of personalized tumors due to their similar size, anatomy, physiology, metabolism, immunity and genetics compared to humans (15–17). Porcine models have already shown to be more predictive of therapeutic responses in humans than murine models (18, 19). Regarding genetics, the pig and human genomes share high homology (20) and both exhibit strongly conserved epigenetic regulation demonstrated by similar genome-wide DNA methylation patterns (21). These characteristics, in addition to recent advances in genetic engineering technologies, makes pigs a robust animal platform for development of suitable genetically defined biological cancer models (17, 22–24).

We have previously developed the transgenic Oncopig Cancer Model using a CreLoxP system containing two mutated genes commonly found in human tumors, the tumor suppressor gene TP53^R167H^ and the oncogene KRAS^G12D^ (25). Several types of cancer have been developed in Oncopigs, including soft tissue sarcoma (25, 26), pancreatic ductal adenocarcinoma (27), and hepatocellular carcinoma (HCC) (28). These studies demonstrate how effective the Oncopig platform is in generating multiple tumor types through induced expression of TP53^R167H^ and KRAS^G12D^ driver mutations. However, urothelial carcinomas have not been developed or studied in this platform to date. Thus, the main objective of this study was to develop novel urothelial carcinoma cell line from Oncopigs and investigate whether these Oncopig bladder cancer cell lines (BCCL) mimic human bladder cancer cell line therapeutic responses *in vitro* following exposure to commercial chemotherapeutic agents (cisplatin, doxorubicin and gemcitabine) commonly used to treat bladder neoplasms clinically.

## 2 Materials and methods

### 2.1 Establishment of Oncopig cancer model urothelial cells

All animal work was performed under an approved University of Illinois Institutional Animal Care and Use Committees (IACUC) protocol. Oncopig urothelial cells (herein called bladder cell line or BCL) were isolated from whole bladders collected from 7 Oncopigs at euthanasia. Whole bladders were collected from each pig and transferred to collection media (HBSS supplemented with NaHCO_3_, 4% penicillin/streptomycin, 2% sterile chlorhexidine) and transported to a tissue culture hood for cell isolation. The bladder was then transferred to a petri dish, where the bladder was drained and turned inside out. The bladder was then placed in a conical tube containing 35 mL of collection media and rocked on a rocker (speed 8) for 15 minutes. The bladder was then washed 4 times by serially dipping into 4 additional conical tubes each containing 35 mL of collection media for 30 seconds each. The bladder was then placed face down on a petri dish, covered in cold collagenase P (1:10 in molecular grade water), and incubated at 37°C for 1 hour. A cell scraper was then used to scrape the inside of the bladder to release cells into the solution. The collagenase P solution containing free floating cells was then transferred to a conical tube and centrifuged at 200 rcf at 4°C for 4 minutes. The media was aspirated, and 30-35 mL of initial growth media (DMEM/F12 supplemented with 10% FBS, 4% penicillin/streptomycin, 100 ng/mL EGF, 0.4 ng/mL Hydrocortisone, and 5 ug/L Amphotericin B) was added to resuspend the cells. Cells were then centrifuged and resuspended an additional 2 times. Cells were then plated into 6 well plates (1x10^5^ cells/well) for culturing (37°C and 5% CO_2_) to establish the primary culture of BCL cells. After 24 hours media was aspirated and replaced with standard growth media (DMEM/F12 supplemented with 10% FBS, 1% penicillin/streptomycin, 10 ng/mL EGF, 0.4 ng/mL Hydrocortisone, and 5 ug/mL Amphotericin B).

### 2.2 AdCre induction

Oncopig BCLs were exposed to Cre recombinase 48 hours post isolation to induce expression of KRAS^G12D^ and TP53^R167H^ transgenes, resulting in the development of Oncopig transformed urothelial cells (herein called bladder cancer cell line or BCCL). Once cells obtained 80% confluency, the standard growth medium was replaced with standard growth medium containing 5% FBS and adenoviral vector encoding Cre recombinase (AdCre; University of Iowa Vector Core, Iowa City, IA) was added at 200 to 400 MOI. Cells were incubated for 5 hrs at 37°C then replenished with fresh medium. Transformed cells were left to grow in the incubator (37°C and 5% CO_2_) to establish BCCL cell lines. After isolation and transformation was performed for each of the 7 animals, one BCCL cell line was chosen at random for *in vitro* experiments (BCCL-2). As all animals were submitted to the same procedures, and cells were isolated and transformed following the same protocols, each cell line is expected to display similar *in vitro* phenotypes and treatment responses.

### 2.3 RT-PCR for genotyping

RNA were isolated from BCL and BCCL cultured cells using the AllPrep RNA Mini Kit (Qiagen, USA) in order to confirm the mutated genes TP53 and KRAS expression after cell transformation using AdCre. Total RNA (1 μg) was reverse transcribed into cDNA in a 20 μl reaction mixture using an Omniscript RT kit (Qiagen, USA) and 1 μl was used in a 25 μl PCR mixture of Hot-StarTaq Plus DNA Polymerase kit (Qiagen, USA). Primers used for amplification of TP53 R167H were 5’-TGGCTCTCCTCAAGCGTATT-3’ and 5’-ATTTTCATCCAGCCAGTTCG-3’. Primers used for amplification of KRASG12D were 5’-TTGTACAGCTAGCTGCTGAAAATGACTGAATAT-3’ and 5’-ATTCTCGAGCGGTTACATAATTATACAC-3’. PCR amplification was performed by 30 cycles of 94°C for 1 min, 94°C for 30 sec, 60°C for 1 min (KRASG12D) or 58°C for 1 min (TP53R167H), followed by a final incubation of 72°C for 10 min. PCR products were analyzed by electrophoresis in an agarose gel.

### 2.4 Immunocytochemistry and immunofluorescence

The experimental methodology involved the seeding of 60,000 BCCL cells onto chambered coverslips with four wells, where they were cultured for 24 hours. Following this incubation, a triple wash with PBS preceded the fixation of cells with 100% methanol. Permeabilization was achieved using 0.25% Triton X-100, followed by a subsequent blocking step utilizing 1% bovine serum albumin (BSA). The immunostaining process included an overnight incubation with anti-Uroplakin II antibody (ab204756, Abcam) at a 1:100 dilution, conducted at 4°C. Post-incubation, cells underwent thorough PBS washes, followed by incubation with anti-rabbit Alexa Fluor 488 secondary antibody (ab150073, Abcam) at a 1:1000 dilution for 1 hour at room temperature. This was succeeded by three consecutive PBS washes, culminating in nuclear staining with DAPI. Cellular observations were carried out using an Olympus BX51 Fluorescence Microscope.

### 2.5 Cell culture

Human bladder cancer cells (5637 and T24 cell lines) were obtained from the Rio de Janeiro cell bank (PABCAM, Federal University of Rio de Janeiro, RJ, Brazil). One Oncopig BCCL was chosen to carry out all *in vitro* experiments. Oncopig BCCL and human T24 cells were cultured in DMEM medium (Vitrocell Embriolife, Campinas, Brazil) and 5637 cells were cultured in RPMI medium. Both mediums were supplemented with 10% fetal bovine serum. The cells grew in a controlled atmosphere at 37°C, 95% humidity and 5% CO_2_. The experiments were performed after cells reached the sub-confluence stage (<90%).

### 2.6 Drug treatment

Cisplatin (solvent: 0.9% aqueous NaCl solution), doxorubicin (solvent: DMSO) and gemcitabine (solvent: H_2_O) were acquired from Sigma-Aldrich and diluted following manufacture instructions. They were stocked at ~4°C protected from light until experiments were performed. At the day of each experiment, the drugs were diluted in media to the desired final treatment concentrations.

### 2.7 Cell proliferation assay (MTT)

5637, T24, and Oncopig BCCL cells were incubated in 96-well culture plates at a density of 2x10^4^ for human cells and 1x10^4^ for porcine cells per well for 24h. These densities were chosen for optimal cell adhesion to the well. Cells were then treated with concentrations of the gemcitabine and cisplatin chemotherapeutic agents ranging from 0.5 to 20 µM and doxorubicin from 0.125 to 5 µM for 48 and 72h hours. Wells containing only culture medium were used as a negative control. After the treatment period, 90 µl of medium plus 10 µl of MTT (tetrazolium [3- (4,5-dimethylthiazol-2-yl) -2,5-diphenyltetrazolium bromide] salt) were added in each well and kept in the incubator for 3 hours at 37°C. Then the absorbance at 492 nm was measured using a spectrophotometer (Microplate Reader MR-96A, Mindray, China). Cell viability (%) was determined using the following equation: Cell viability = {100-[(1- Abs^492^treated cells/Abs^492^control cells) x 100]}.

### 2.8 Confocal microscopy

T24 and 5637 cells were seeded at a density of 2x10^4^ and Oncopig BCCL at 1x10^4^ in 96-well plates. After 24h, treatment was administered using concentrations of 0.5, 1, and 5 µM for gemcitabine, 5, 10, and 20 µM for cisplatin, and 0.25, 0.5, and 1 µM for doxorubicin for 48h. After the treatment period, staining with either Live/Dead or DAPI assay was applied, and cellular analysis was performed in the confocal microscope (Leica Microsystems) for cell visualization and integrity analysis.

#### 2.8.1 Live/dead assay

To obtain cytotoxicity data, a Live/Dead assay was performed as indicated by the manufacturer (ThermoFisher Scientific). Briefly, cells were washed with PBS and then stained with Calcein AM and ethidium homodimer-1 (EthD-1) for 30 min in the incubator in the dark. Soon after, cells were washed two more times. Photos of three distinct fields were obtained from each well and the number of green and red cells was later counted with the Cell ^ F (Olympus) program.

#### 2.8.2 DAPI assay

Knowing that chemotherapies usually cause cell death through apoptotic pathways, a DAPI assay was applied to obtain the percentage of apoptotic cells after treatments. DAPI ((4′,6-Diamidino-2-phenylindole dihydrochloride) is a nuclear fluorophore that stains the DNA of all cells blue, but apoptotic cells display a greater fluorescence due to its compressed DNA and higher membrane permeability. Briefly, cells were washed with PBS and permeabilized using a solution of acetone and methanol (1:1) for 5 minutes and then washed again two times before the DAPI assay (ThermoFisher Scientific) was applied at room temperature for 5 minutes, following the manufacturer’s instructions. Images were acquired with a Leica Confocal Microscope (Leica Microsystems). Photos of three distinct fields were obtained and were later analyzed using the software ImageJ (nih.gov). Apoptosis induction (%) was obtained using the following equation: (apoptotic cells\total number of cells) x 100. The control group consisted of cells exposed to medium only.

### 2.9 Via count

For the ViaCount assay, cells were seeded in 12-well plates at a density of 5x10^4^ cells per well. After 24h, the following treatments were administered: gemcitabine at 1µM and 5 µM, cisplatin at 10 µM and 20 µM, and doxorubicin at 0.5 µM and 1 µM. Cells were then harvested, centrifuged, and resuspended in 50 μL of medium plus 450 μL of ViaCount reagent (Guava Technologies) according to the manufacturer’s instructions, obtaining a final concentration of >10^5^ cells/mL. Samples were read on the Muse Cell Analyzer cytometer. The control group consisted in cells exposed to medium only.

### 2.10 RNA isolation for gene transcription analysis

RNA extractions of human and Oncopig cells were performed in 6-well plates at a density of 2x10^5^ cells per well after exposure to the chemotherapeutic gemcitabine at 1 µM. The control group consisted of cells exposed to medium only. After the treatment period of 48h, cells were washed with PBS and total RNA from the samples was extracted using TRIzol reagent, following manufacturer instructions (Invitrogen). Then, Qiagen RNeasy Kits (QIAGEN GmbH, Hilden, Germany) were used to isolate and purify the RNA according to manufacturer’s recommendations. The quality and integrity of total RNA was verified by Nanovue Spectrophotometer (GE Healthcare) and Agilent 2100 bioanalyzer kit (Agilent Technologies, Santa Clara, CA, USA).

#### 2.10.1 cDNA synthesis

cDNA synthesis was performed using 10 µl of total RNA at 100 ng/µl added to 10 µl of the High Capacity cDNA Reverse Transcription kit (Applied Biosystems ™, UK). The reaction condition was 25°C for 10 minutes, 37°C for two hours, 85°C for 5 minutes and 4°C to finish the reaction, followed by storage of the samples at –20°C.

#### 2.10.2 Microarray assay

To obtain the gene transcription panel of treated and untreated human and Oncopig cells, the Two-Color Microarray-Based Gene Expression Analysis microarrays (Agilent Technologies Inc, Santa Clara, CA, USA) were used according to the manufacturer’s instructions. Samples of treated and untreated 5637 and Oncopig BCCL cells were used in this experiment. Microarray slides “HD Human GE 4 × 44K v2 Microarray” (1 slide, part number: G2519F-026652) and “Porcine (V2) Gene Expression Microarray, 4 × 44K” (1 slide, part number: G2519F-026440) were used for hybridization. Microarray data is available at the Gene Expression Omnibus Archive (GEOarchive) under accession number (GSE255187).

#### 2.10.3 Microarray data analysis

Microarray slide analysis was performed in the software GeneSpring GX (Agilent Technologies Inc, Santa Clara, CA, USA). Untreated and treated groups of each cell line were first compared to each other using p (<0,05) and fold change to identify differentially expressed genes (DEGs) (2). DEGs were then compared between the human and Oncopig samples to identify common transcriptional changes in response to gemcitabine exposure.

### 2.11 Cytoskeleton reorganization

5637 and Oncopig BCCL cells were plated in 96-well plates. After 24h, 1µM of gemcitabine was administered into the cells for a 48h period. Then, two washes with PBS were made and fresh medium was added. Twenty-four hours later, new washes were applied before cells were fixed with formaldehyde 3.7% and permeabilized with triton x-10 0.1%. Cell nucleus was then stained with DAPI (Invitrogen, USA) and the cytoskeleton with Texas Red (Invitrogen, USA) according to manufacturer’s instructions. Images were acquired using confocal microscopy (Leica Microsystems) in the XYZ mode to capture all the length of the cell.

### 2.12 *In silico* studies

We used *in silico* studies to compare similarities between human and swine proteins related to the metabolism of bladder cancer chemotherapeutics (doxorubicin, gemcitabine and cisplatin). The porcine amino acid (AA) FASTA sequences were obtained in the NCBI Protein website (https://www.ncbi.nlm.nih.gov/protein/). The human FASTA sequence was retrieved from the Protein Data Bank (https://www.rcsb.org/). The proteins analyzed were: Deoxycytidine Kinase (DCK, PDB:2QRO), Cytochrome P450 3A4 (CYP450, PDB:5A1R), balancer nucleoside transporter 1 (ENT1 or SLC29A1, PDB:6OB6), UMP-CMP Kinase (CMPK1, PDB: 1TEV), Aldo - Ket A1 family member 1 reductase (AKR1A1, PDB: 2ALR), NAD(P)H Dehydrogenase [quinone] 1 (NQO1, PDB:5FUQ), and Phosphoinositide 3-kinase (PI3K, PDB: 7MEZ).

We used the Basic Local Alignment tool (BLAST) from NCBI to analyze the similarity between the protein species (https://blast.ncbi.nlm.nih.gov/Blast.cgi). Then, the FASTA sequence alignment was performed using the Software clustal Omega (https://www.ebi.ac.uk/Tools/msa/clustalo/). Furthermore, we performed homology modeling of porcine and murine proteins using the human protein as the model in the software SWISS - Model to obtain the 3D structure of these proteins (https://swissmodel.expasy.org/). The human 3D crystalized proteins were obtained from the Protein Data Bank repository.

Finally, we performed molecular docking of the enzymes cited above to predict the interaction between them and their ligands (cisplatin, doxorubicin and/or gemcitabine), using the software Autodock Tools 4.2. We optimized the crystalized proteins with Autodock software Tools 4.2., and the removal of binders that came with the files was performed by the software Discovery Studio 2020. The drug structure of Cisplatin (DrugBank Code: DB00515), Gemcitabine (Drugbank Code: DB00441) and Doxorubicin (Drugbank Code: DB00997) was obtained from the DrugBank database. Interactions between the drugs gemcitabine and doxorubicin were visualized by Discovery Studio 2020 software. For cisplatin, the PLIP program was used due to a limitation of Discovery Studio 2020, which is not able to recognize Cisplatin as a ligand.

### 2.13 Statistical analysis

Statistical analysis was performed with Graphpad Prism 5 software using Two-Way ANOVA. A Bonferroni post-test was applied. P <0.05 was considered statistically relevant. IC^50^ was calculated in the GraphPad software as well to determine the drug concentration required to inhibit 50% of cellular growth. All experiments were performed in triplicate.

## 3 Results

### 3.1 Oncopig BCCLs recapitulate human bladder carcinoma cells therapeutic responses

First, RT-PCR was applied after several passages to access TP53^G12D^ and KRAS^R167H^ transcription in cells prior to (BCL) and after (BCCL) AdCre exposure. BCCL cells expressed TP53^G12D^ and KRAS^R167H^, while BCL was did not (**Supplementary Figure 1A**), indicating that mutated genes were only expressed upon CRE recombinase exposure. In addition, BCCL cells stained positive for Uroplakin II, a marker for urothelial carcinoma (**Supplementary Figure 1B**).

The ability of biological models to mimic therapeutic responses observed in humans is critical in the search for more suitable models. Therefore, we aimed to investigate if Oncopig BCCLs mimic human urothelial carcinoma cell (5637 and T24) responses to commercial chemotherapeutic agents used to treat bladder neoplasms clinically. We chose three of the most commonly used drugs in bladder cancer treatment: cisplatin, doxorubicin and gemcitabine.

**Figure 1:**
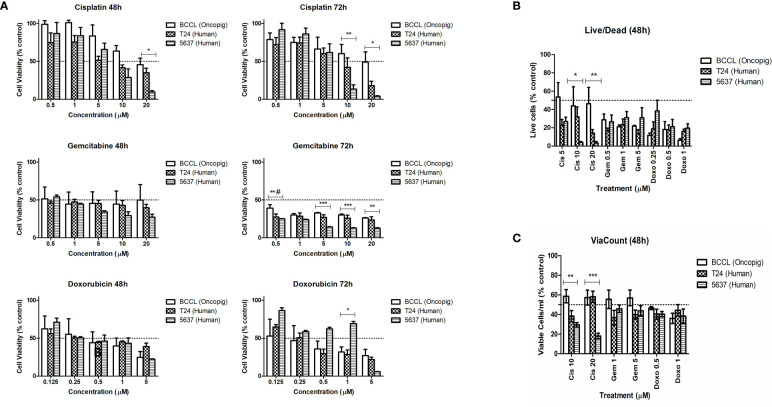
Human and Oncopig cell line sensitivity to chemotherapeutics. Human bladder cancer cell lines (5637 and T24) and Oncopig BCCLs treated with commercial chemotherapeutics cisplatin, gemcitabine, or doxorubicin. **(A)** Antiproliferative activity in cells treated for 48 and 72h obtained by MTT assay. Y axis: Cell viability (relative to % of each cell line control). **(B)** Cytotoxicity results obtained by Live/Dead assay. Y-axis: number of viable cells (% of control). **(C)** ViaCount assay results. Y-axis: number of viable cells/mL (% of control). All data are expressed as mean ± SEM of three independent experiments. Asterisks (*) indicate significant differences between Oncopig BCCL and 5637 means and hashtag (#) indicates significant differences between Oncopig BCCL and T24 means at the same drug concentration. * or #= p<0.05. **= p<0.01. ***= p<0.001. The Y axis dotted line represents 50% of growth inhibition.

We started by using five different concentrations of each drug to first establish their IC^50^ value. Similar responses were observed for Oncopig and human bladder cancer cells after treatment with cisplatin, gemcitabine, and doxorubicin (**Figure 1A**), translating to similar IC^50^ values between the three cell lines (**Table 1**). Like T24 and 5637 cells, Oncopig BCCLs were extremely sensitive to doxorubicin and gemcitabine. The IC^50^ for Oncopig BCCLs in response to doxorubicin treatment was 0.3966 ± 0.8µM and 0.2128 ± 0.2 µM at 48h and 72h, respectively. T24 cells displayed nearly the same IC^50^ values (0.3875 ± 0.2 µM at 48h and 0.2461 ± 0.09 µM at 72h). On the other hand, 5637 cells were slightly more resistant to doxorubicin treatment, with IC^50^ values of 0.4339 ± 0.15 µM and 0.925 ± 0.4 µM at 48h and 72h, respectively (**Table 1**), which is still considered highly sensitive to the drug. Statistical differences in doxorubicin treatment response were only identified when exposing cells to 1µM for 72h (**Figure 1A**). Human and Oncopig cells treated with gemcitabine for 48h had no statistical difference. Following 72h of treatment, T24 human cells also demonstrated similar results compare to Oncopig BCCLs. In contrast, there were some punctual differences between the Oncopig BCCL and 5637 human cells. In addition, Oncopig BCCLs proved to be more resistant to cisplatin when compared to both human cell lines (**Figure 1A**), requiring a 3x higher dose to reach 50% of cell inhibition (**Table 1**). There were statistical differences between 5637 and Oncopig BCCLs at 10 and 20 µM, but not T24.

**Table 1:** Oncopig and human cell line sensitivity to chemotherapeutics.

Doxorubicin	IC^50^ 48h	IC^50^ 72h
**BCCL**	0.3966 ± 0.8 µM	0.2128 ± 0.2 µM
**T24**	0.3875 ± 0.2 µM	0.2461 ± 0.09 µM
**5637**	0.4339 ± 0.15 µM	0.9258 ± 0.4 µM
Gemcitabine	IC^50^ 48h	IC^50^ 72h
**BCCL**	1.647 ± 4.8 µM	0.3308 ± 0.2 µM
**T24**	1.242 ± 1.3 µM	0.1787 ± 0.2 µM
**5637**	0.9881 ± 0.5 µM	0.1208 ± 0.06 µM
Cisplatin	IC^50^ 48h	IC^50^ 72h
**BCCL**	15.73 ± 4.2 µM	11.01 ± 8.2 µM
**T24**	5.027 ± 2.3 µM	4.631 ± 2.4 µM
**5637**	5.841 ± 2.15 µM	5.643 ± 1 µM

IC^50^ ± SD values for doxorubicin, gemcitabine, and cisplatin exposure for 48 and 72h in Oncopig BCCL, T24, and 5637 cells. IC^50^ is a quantitative measure that indicates the concentration needed to inhibit cell proliferation by 50%. Values were obtained using GraphPad Prism 5 and are representative of three independent experiments.

**Figure 2:**
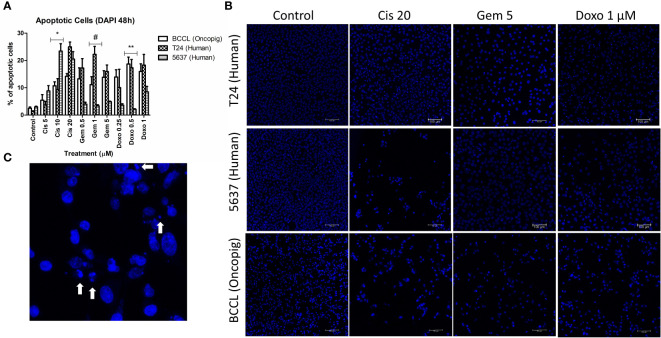
Apoptosis induction in Oncopig and human cell lines after drug treatment. Human bladder cancer cell lines (5637 and T24) and Oncopig BCCLs treated with commercial chemotherapeutics cisplatin, gemcitabine, or doxorubicin for 48h and subjected to DAPI assay. **(A)** Apoptosis induction percentage. Asterisks (*) indicate significant differences between Oncopig BCCL and 5637 means and hashtag (#) indicates significant differences between Oncopig BCCL and T24 means at the same drug concentration. * or #= p<0.05. **= p<0.001. Y axis: Apoptosis induction percentage. X axis: drug treatment. **(B)** Confocal microscopy images. Blue represents cell nucleus staining (emission ~460 nm). White arrows indicate apoptotic cells that are distinguished by their higher fluorescence and condensed DNA.

The IC^50^ concentrations were used to further investigate Oncopig and human bladder cancer cell line cytotoxic responses. Results from Live/Dead and ViaCount assays demonstrated a similar cytotoxic response between human and Oncopig cell lines following treatment with cisplatin, gemcitabine, and doxorubicin (**Figures 1B, C**; **Supplementary Figure 2**). Again, statistically significant differences were identified between 5637 and Oncopig BCCLs for cisplatin treatment at 10 and 20 uM (**Figures 2B, C**) mainly because 5637 was highly sensitive to cisplatin at these concentrations. Our findings are consistent with previous studies demonstrating that 5637 cells are more sensitive to cisplatin treatment than T24 cells (29–32). Taken together, all three cell lines were extremely sensitive to the tested chemotherapeutics, displaying a dose-dependent cytotoxicity (**Figures 1A–C**). It is important to highlight that differences in treatment response were observed between the two human cell lines as well (T24 and 5637; statistical data not shown).

Previous studies have demonstrated that a wide range of chemotherapeutic agents provoke cell death by apoptosis induction (33–35). Therefore, we aimed to investigate if Oncopig BCCLs would behave similarly to human bladder cancer cells by triggering apoptosis after drug exposure. An increase in apoptotic cells was observed after treatment with cisplatin, gemcitabine, and doxorubicin in both human and Oncopig cell lines. Once again, Oncopig BCCL cellular responses were more closely related to T24 cells. 5637 cells demonstrated lower levels of apoptosis after doxorubicin and gemcitabine treatment when compared to Oncopig BCCLs and T24 (**Figure 2**).

### 3.2 Oncopig and human bladder cancer cells display consistent modulation of cytoskeleton and apoptotic gene transcription following gemcitabine treatment

After cytotoxic results were obtained, microarray analysis was carried out to monitor genome-wide expression changes and identify gene transcription patterns altered following gemcitabine treatment to gain new insights into the similarities between human and Oncopig cancer cell responses at the molecular level. 5637 and Oncopig BCCLs were compared due to their similar responses to gemcitabine treatment, focusing on genes displaying consistent up or down regulation in both lines. Two main processes were found to be altered in both cell lines following gemcitabine treatment: apoptosis and cytoskeleton organization (**Table 2**).

**Table 2:** Most relevant DEGs common between Oncopig and human cell lines in response to gemcitabine treatment.

Cellular Process	GENE	Function	Human	Oncopig
**Apoptosis**	BCL-2	Anti-apoptotic	Up	Up
**Apoptosis**	TP53INP1	Stress inducible protein	Up	Up
**Apoptosis**	PARM1	Resist apoptosis	Up	Up
**Apoptosis**	GADD45B	Response to environmental stress such as treatment with DNA-damaging agents	Up	Up
**Cytoskeleton**	ODF2	Outer dense fiber proteins	Up	Up
**Cytoskeleton**	ZMYM6	Regulation of cell morphology and cytoskeletal organization	Down	Down
**Cytoskeleton**	CKAP2	Cytoskeleton-associated protein involved in mitotic progression	Up	Up
**Cytoskeleton**	ACTA-2	Actin protein isoform	Up	Up
**Cytoskeleton**	ACTB	Actin protein isoform	Down	Down
**Cytoskeleton**	ARHGAP21	GTPase-activating protein (GAP). Regulates F-actin dynamics	Down	Down
**Cytoskeleton**	RND1	Signaling G protein. Is a part of the GTPases family.	Up	Up
**Cytoskeleton**	SYNM	Intermediate filament family member	Up	Up
**Cytoskeleton/bladder cancer marker**	FGFR3	Fibroblast growth factor receptor 3	Up	Up

Differences in expression are expressed as changes in treated compared to control groups (cells exposed only to medium).

Surprisingly, the known anti-apoptotic protein BCL-2 was upregulated in Oncopig and human cells after treatment (**Table 2**) while no difference in expression of the pro-apoptotic protein BAX was observed in either cell type after gemcitabine treatment (data not shown), indicating that apoptosis was not being activated through the Bax/Bcl-2 proteins. These proteins are regulated by the tumor suppressor gene TP53, which was downregulated in the 5636 cells while no transcriptional difference was observed in Oncopig BCCLs after treatment. Results from Saalfrank et al. (2016) in transformed pig cells with a latent oncogenic TP53^R167H^ mutation previously demonstrated increased TP53 expression due to TP53^R167H^ oncogene activation. The mutant expression affected downstream genes such as BAX expression (36). Therefore, the lack of difference in TP53 expression in Oncopig BCCLs may be due the transgenic TP53^R167H^ mutation being expressed in a similar matter in both treated and untreated Oncopig cells. Further, MDM4, a protein that binds the p53 protein and inhibits its activity (37), was over-expressed in Oncopig BCCLs following gemcitabine treatment (**Table 3**). All these findings help confirm that gemcitabine treatment does not trigger the p53/Bax/Bcl-2 mitochondrial pathway in both cell lines.

**Table 3:** Most relevant DEG discrepancies between Oncopig and human cells in response to gemcitabine treatment.

Cellular Process	GENE	Function	Human	Oncopig
**Apoptosis**	Caspase 3	Execution of apoptosis	Up	No difference
**Apoptosis**	Caspase 10	Execution of apoptosis	Up	No difference
**Apoptosis**	CASP8AP2	Regulatory role in Fas-mediated apoptosis	Up	No difference
**Apoptosis**	TP73	Member of the P53 family of transcription factor. Apoptosis response to DNA damage	Up	No difference
**Apoptosis**	TP53	Tumor suppressor gene. Apoptosis response to DNA damage	Down	No difference
**Apoptosis**	FAS	Member of the TNF-receptor superfamily. Physiological regulation of programmed cell death	Up	No difference
**Apoptosis**	Rb1	Tumor suppressor gene. Negative regulator of the cell cycle	Up	No difference
**Apoptosis**	MDM4	Inhibits Tp53 and Tp73-mediated cell cycle arrest and apoptosis	No difference	Up

Differences in expression are expressed as changes in treated compared to control groups (cells exposed only to medium).

**Figure 3:**
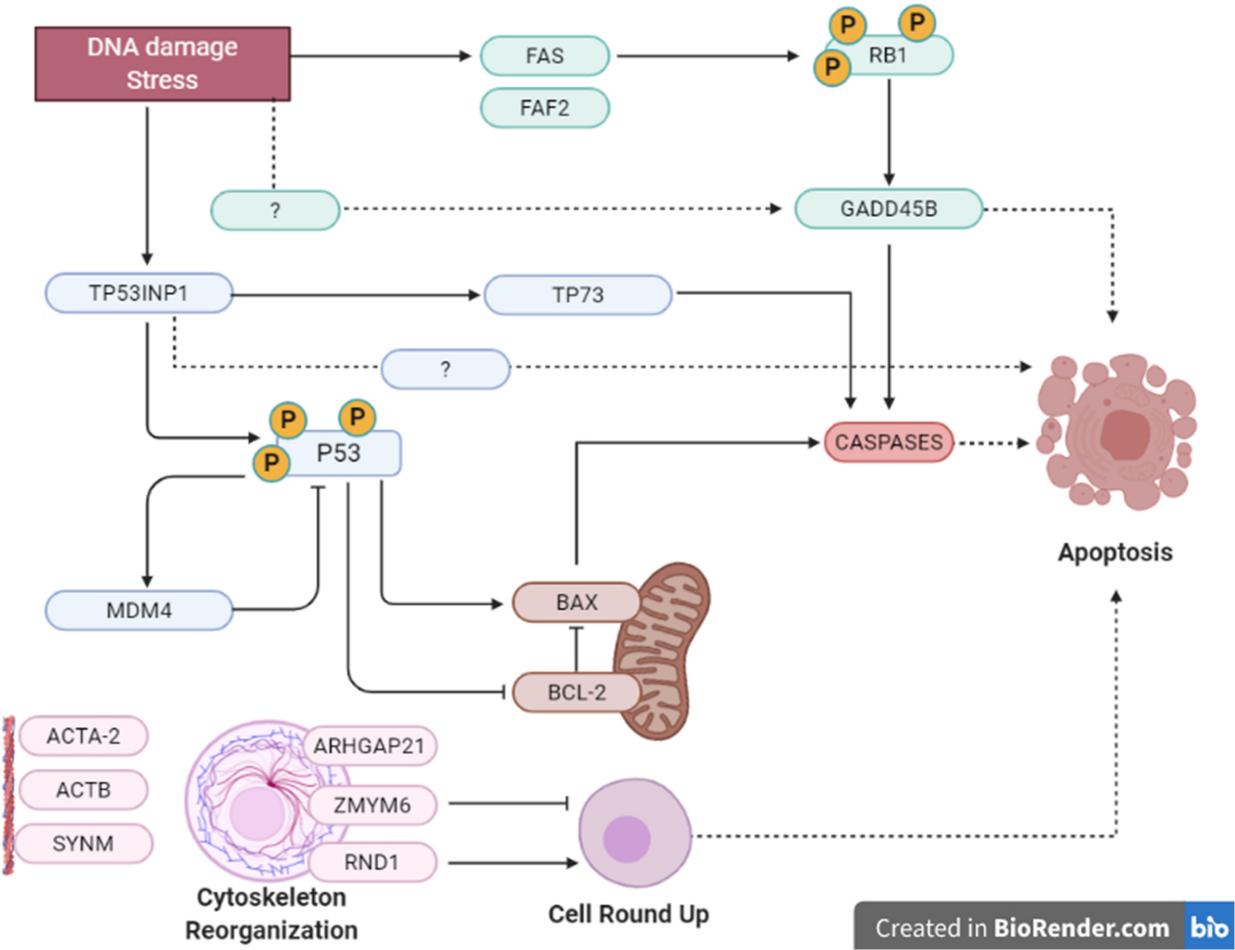
Summary of apoptosis pathways in human and Oncopig cells. Summarized apoptotic pathways altered by gemcitabine treatment in Oncopig BCCL and 5637 cells evaluated by microarray. There were multiple possible cellular pathways involved in apoptosis induction, such as: TP53INP1/P53/BCL/BAX/CASPASE mitochondrial intrinsical pathway; TP53INP1/TP73 activation; FAS/RB1/GADD45B/CASPASE pathway and cell rounding up by RND1 and/or ZMYM6.

Nonetheless, genes related to apoptosis induction, such as TP53INP1 and GADD45B, were upregulated after gemcitabine treatment in both cell lines (**Table 2**). Tumor protein 53-induced nuclear protein 1 (TP53INP1) is a stress-induced p53-target gene with an important role in cellular homeostasis and DNA damage response due to its anti-proliferative and pro-apoptotic activity (38). TP53INP1 is usually regulated by TP53. However, it has been shown that in the absence of p53, TP53INP1 transcription can also be induced by p73, a p53 homolog, which has been demonstrated to be up-regulated in response to other DNA-damaging agents, such as cisplatin and gamma-irradiation (39, 40) (**Figure 3**). Our study found that both TP53INP1 and P73 were up-regulated in 5637 cells after gemcitabine treatment, however, only TP53INP1 was up-regulated in Oncopig BCCLs (**Table 3**).

In addition, GADD45B (Growth Arrest and DNA Damage protein 45b) is a known positive mediator of apoptosis that has been implicated in stress signaling in response to physiological or environmental stressors (41). GADD45B was upregulated in 5637 and Oncopig BCCLs after gemcitabine exposure (**Table 2**). In murine hepatocytes, GADD45B plays a role in the Fas-induced apoptosis by mediating p38-induced Rb phosphorylation and enhancing the interaction between these two proteins (42) (**Figure 3**). Herein, both FAS and RB1 genes were upregulated in 5637 cells after gemcitabine exposure, in addition to caspases 3, caspase 10, and CASP8AP2 (**Table 3**). This is consistent with previous findings demonstrating that 5637 apoptotic responses to gemcitabine treatment include caspase (3, 7 and 9) activation through Fas overexpression while expression of genes related to the Bcl-2/Bax pathway are not affected, indicating that the Fas pathway is more likely to be activated (43). Interestingly, Oncopig BCCLs demonstrated upregulation in the FAF2 gene (Fas associated factor family member 2) but not FAS, RB1, or caspase genes following treatment with gemcitabine (**Table 3**). Other study using TP53^R167H^ and KRAS^G12D^ transformed pig cells’ found similar results demonstrating the mutant TP53^R167H^ capacity to harm MDM2 downstream genes such as FAS, BAX, and CASP6 (36). Fas-mediated apoptosis in response to stress-inducing and DNA-damaging agents is still poorly understood in swine. Therefore, deeper investigation towards the role of fas-related proteins in apoptotic swine cells needs to be conducted.

**Figure 4:**
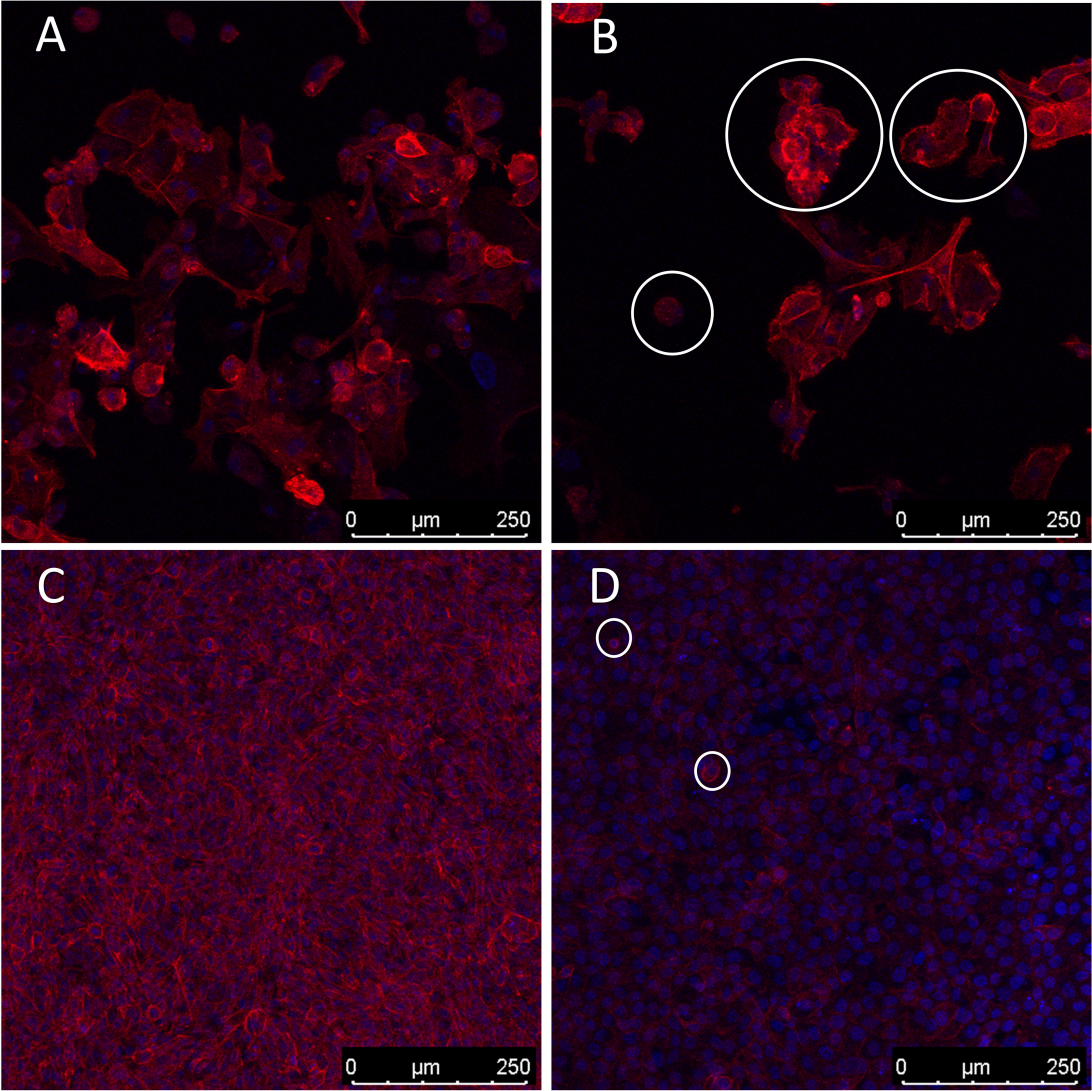
Cytoskeleton reorganization. Blue represents cells’ nucleus staining with DAPI (emission ~460 nm). Red represents cytoskeleton staining with Texas Red (emission ~615 nm). 200x magnification. White circles demonstrate examples of cell rounding. **(A, B)**: Oncopig cells (BCCL). **(C, D)**: human cells (5637). **(A, C)**: untreated cells. **(B, D)**: cells treated with 1µm of gemcitabine.

Moreover, it is well known that cells undergoing apoptosis experience certain hallmarks, including actin reorganization (44). Herein, we highlight the cytoskeleton role in apoptosis induction (45, 46). Actin cytoskeleton has been reported as an important mediator and initiator of apoptosis signaling where dramatic cellular changes in actin filament organization and cellular morphology can be evidenced in different stages of apoptosis (45). Our microarray analysis demonstrated altered transcription in both human and Oncopig cells in genes related to cytoskeleton proteins, such as beta actin (ACTB), alpha actin (ACTA2), CKAP2, SYNM, and ODF2, as well as genes involved in actin and/or cytoskeleton organization, like ARHGAP21, RND1, and ZMYM6 (**Table 2**). Among them, we highlight ZMYM6 and RND1, which have been previously investigated for their role in cellular rounding. Cells undergoing apoptosis tend to round up and detach from their surroundings due to loss of cellular attachment/anchorage with the extracellular environment. This process is known as anoikis, a special type of apoptosis (47). Studies using siRNA in Drosophila cells demonstrated that down-regulation of ZMYM6 caused cellular rounding up (48). Herein, ZMYM was down regulated in both arrays and this inactivation could be leading to cellular round up and consequently apoptosis. In addition, RND1 is a part of the Ras superfamily of small GTPases, which have been shown to control the actin cytoskeleton. RND1 transcription induces loss of focal adhesions to the extracellular matrix due to cell body round up, causing the cell to lose all adhesion and detach (49) (**Figure 4**). Our results showed that RND1 was upregulated in both human and Oncopig cells, likely resulting in cellular round up and apoptosis. Texas Red staining also demonstrated cells treated with 1µM of gemcitabine reorganized their cytoskeleton to a more rounding shape (**Figures 4A–D**). It was observed that the cytoskeleton became less evident and protruding in treated cells (**Figure 4D**). Taken together, our findings support the theory that Oncopig and human bladder cancer cells undergo apoptosis through the same molecular mechanisms in response to gemcitabine treatment. This is supported by the fact that both human and Oncopig cells display a lack of p53/Bax/Bcl-2 pathway activation, increased transcription of stress-related-apoptotic genes such as GADD45B and TP53INP1, and reorganize cytoskeleton/actin to trigger apoptosis upon gemcitabine treatment.

### 3.3 Porcine, human, and murine enzymes show similar interactions between chemotherapeutics and their target proteins *in silico*

BLAST results demonstrated that proteins CYP450, ENT1, AKR1A1, DCK, CMPK1, NQO1, and PI3K are highly similar (>70%) between pigs and humans, with pig/human protein homology ranging from **76.15%** to up to **97.4%**. Mouse/human homology ranged from **71.8%** to **98.5%** (**Table 4**). After successfully obtaining the swine 3D proteins by homology modelling using the human proteins as a reference, we performed molecular docking to verify if gemcitabine, doxorubicin, and cisplatin would interact with the active site of human, porcine, and murine enzymes involved in the metabolism of each of these drugs in a similar way. Our results indicated that all species had similar interactions (Δg values) between the chemotherapeutics and their target proteins (**Table 5**).

**Table 4:** Human, pig, and mouse protein homologies.

Protein	Pig/Human similarity	Mouse/Human similarity
**DCK**	88.08%	93.5%
**CYP450**	76.15%	71.8%
**ENT1**	86.6%	79.3%
**CMPK1**	97.4%	98.5%
**AKR1A1**	94.2%	93.2%
**NQO1**	85.8%	86.5%
**PI3K**	95.19%	84.2%

Result of similarities between human, porcine, and murine proteins obtained using BLAST. These proteins are involved in the metabolism of chemotherapeutics cisplatin, doxorubicin, or gemcitabine.

**Table 5:** Human, pig, and mouse activation energies.

Protein	ΔG (kcal/mol) Human	ΔG (kcal/mol) Swine	ΔG (kcal/mol) Murine	Chemotherapy drug
**CYP450**	**-6.68**	**-6.44**	**-6.59**	**gemcitabine**
**CYP450**	**-12.26**	**-11.87**	**-12.44**	**doxorubicin**
**CYP450**	**-5.99**	**-4.91**	**-7.3**	**cisplatin**
**DCK**	**-6.98**	**-6.88**	**-6.47**	**gemcitabine**
**ENT1**	**-6.29**	**-5.86**	**-6.29**	**gemcitabine**
**CMPK1**	**-5.27**	**-5.28**	**-5.68**	**gemcitabine**
**AKR1A1**	**-11.75**	**-10.35**	**-10.19**	**doxorubicin**
**NQO1**	**-9.52**	**-9.09**	**-9.98**	**doxorubicin**
**PI3K**	**-7.71**	**-6.01**	**-6.72**	**cisplatin**

Values referring to the activation energy in kcal/mol (Δg) of each protein and its respective ligand (chemotherapeutic agent).

We obtained an activation energy (Δg) prediction of -6.68, -6.44, and -6.59 between gemcitabine and human, porcine, and murine CYP450 proteins, respectively. CYP450 and doxorubicin showed a Δg of -12.26, -11.87, and -12.44 with the human, porcine, and murine proteins, respectively, while CYP450 and cisplatin demonstrated a Δg of -5.99, -4.91, and -7.3 between the same species, respectively (**Table 5**). Regarding gemcitabine and DCK, CMPK1, and ENT1, the interaction generated a Δg of -6.98, -6.88, and -6.47 for DCK, -6.29, -5.86, and -6.29 for ENT1, and a Δg of -5.27, -5.28, and -5.68 for CMPK1 for human, porcine, and murine proteins, respectively (**Table 5**). In addition, we obtained a Δg of -11.75, -10.35, and -10.19 for the interaction between doxorubicin and AKR1A1 (**Table 5**), and -9.52, -9.09, and -9.98 for NQO1 for human, porcine, and murine proteins, respectively. Finally, the interaction between human, porcine, and murine PI3K and cisplatin generated a Δg of -7.71, -6.01, and -6.72, respectively (**Table 5**). The residues responsible for the protein-ligand interactions are shown in **Supplementary Table 1** and **Supplementary Figures 3**-**11**.

## 4 Discussion

The Oncopig is a genetic porcine model capable of generating tumors through the activation of TP53^R167H^ and KRAS^G12D^ driver mutations, which are found in ⅓ (50) and ¼ (51) of all human cancers, respectively. Several tumor types including soft tissue sarcoma (25, 26), pancreatic ductal adenocarcinoma (27) and HCC (28) have been successfully modelled in this platform. However, urothelial carcinoma has not yet been induced in the Oncopig. Herein, as a proof of concept to demonstrate that urothelial cells derived from Oncopigs can be transformed *in vitro*, we isolated for the first time cells from the Oncopig bladder. These cells were transformed *in vitro* through expression of the TP53^R167H^ and KRAS^G12D^ driver mutations, as previously reported (25, 27, 28).

Biological models must mimic the human cellular response to therapies to succeed in translating preclinical results to clinical practice. Hence, we aimed to investigate if these novel Oncopig urothelial carcinoma cells would mimic human urothelial carcinoma cellular (5637 and T24) responses to commercial chemotherapeutic agents used to treat human urothelial carcinoma. Herein we confirmed that Oncopig and human urothelial carcinoma cells display similar cytotoxic responses and apoptosis induction *in vitro* when exposed to chemotherapeutics (gemcitabine, doxorubicin and cisplatin). Several studies report high sensitivity of 5637 and T24 cells to gemcitabine, doxorubicin, and cisplatin (29–32). Likewise, Oncopig BCCLs were also extremely sensitive to these drugs.

Finally, porcine, mouse, and human proteins were demonstrated to interaction with the drugs cisplatin, doxorubicin, and gemcitabine *in silico* in a similar matter, evidenced by almost identical Δg values, further indicating that porcine models would metabolize these chemotherapeutics like humans, and should complement results obtained in mouse models. DCK is a key enzyme that activates gemcitabine through phosphorylation (52). ARG128 residue plays a role in anchoring compounds in the enzyme and interacting with gemcitabine (53), and both human and pig proteins displayed that interaction *in silico* in our results. The ENT1 protein, a member of the SLC29 family, is the most abundant nucleoside transporter located in the plasma membrane and distributed in human cells, whose one of the functions is to mediate the entry of antineoplastic drugs derived from nucleosides (54). Two matching residues were found in porcine and human interaction with gemcitabine, GLY154 and GLN158. The first one is a possible determinant of the specificity in the binding of compounds with ENT1, while GLN158 is important in the recognition of nucleobases, known as nitrogenous bases, such as gemcitabine (55), which could indicate a similar compound uptake in both species.

Next, after confirming our theory that Oncopig BCCLs mimic human cellular responses to chemotherapeutics *in vitro*, we further investigated similar transcriptional changes between Oncopig and human urothelial carcinoma cells following gemcitabine treatment. We identified two main cellular processes altered in both 5637 and Oncopig BCCLs: apoptosis and cytoskeleton organization. These two processes are actually linked, as cells undergoing apoptosis usually suffer cytoskeleton reorganization (45, 46). Based on our findings, we believe that apoptosis induction in human 5637 and Oncopig BCCLs after gemcitabine exposure was triggered by up regulation of RND1 and down regulation of ZMYM, inducing cellular rounding up and subsequent detachment of cells from the culture flask (48, 49). In addition, our results demonstrate upregulation of genes related to stress-induced-apoptosis in both human and Oncopig cells, including GADD45B (41) and TP53INP1 (38), that may also be involved in the analogous cytotoxic response of these cells following gemcitabine treatment.

This work is not without flaws. As a preliminary study, we initially only studied apoptotic pathways since it is the main pathway triggered by the tested chemotherapeutics (33–35). Our microarray results also indicated an involvement of apoptosis process in treated cells. Yet, tumor cell death is a complex process involving several mechanisms. Therefore, the lack of additional *in vitro* cell death pathways comparisons beyond apoptosis is a limitation that needs to be addressed in future studies. In addition, one Oncopig BCCL cell line was chosen at random for *in vitro* experiments. As all animals were submitted to the same procedures, and cells were isolated and transformed following the same protocols, each cell line is expected to display similar *in vitro* phenotypes and treatment responses. However, this is also a limitation of the present work since differences between pig BCCL cells’ drug responsiveness cannot be completely ruled out. In addition, it is important to note that while this study represents an initial demonstration of the potential translational relevance of the Oncopig bladder cancer model, further *in vitro* and *in vivo* investigations are required to confirm the model’s potential. As, Porcine Gene Expression Microarray slide (44K) annotations are yet to be completed, some important pathways may not have been identified due to a lack of information for several genes in the porcine array. Finally, microarray analysis was not performed for T24 cells or following cisplatin treatment due to limited funding. Comparison of Oncopig BCCL and 5637 response to gemcitabine was selected due to the high similarities in response (no statistically significant differences observed). Comparative analysis of other cell lines and treatments are required as part of future studies.

## 5 Conclusions

In conclusion, our results indicate that the Oncopig Cancer Model is an attractive animal to model urothelial carcinoma due to its similar *in vitro* therapeutic responses compared to human bladder cancer cells. Oncopig BCCLs could therefore serve as an *in vitro* screening platform. In addition, this work contributes to the characterization and scientific acceptance of the Oncopig as an appropriate biological model to study cancer, more specifically bladder cancer. The Oncopig can play an important role in drug screening of cancer therapies by providing a model more predictable of therapeutic responses. Finally, the Oncopig could serve as a translational drug testing platform following *in vitro* screening of compounds and subsequent testing on small animal models (for example, rodents) in order to prove the effectiveness of therapy before advancing to expensive clinical trials in humans. As this is a preliminary study, further work is needed to confirm if Oncopig and human urothelial carcinoma display similar histological and molecular features, and to develop strategies for *in vivo* Oncopig urothelial carcinoma tumor development.

## Data availability statement

The original contributions presented in the study are publicly available. This data can be found here: Gene Expression Omnibus Archive (GEOarchive), accession number GSE255187.

## Ethics statement

Ethical approval was not required for the studies on humans in accordance with the local legislation and institutional requirements because only commercially available established cell lines were used. The animal study was approved by University of Illinois at Urbana-Champaign IACUC. The study was conducted in accordance with the local legislation and institutional requirements.

## Author contributions

NS: Formal analysis, Investigation, Writing – original draft, Writing – review & editing. LS: Formal analysis, Investigation, Writing – review & editing. CB: Formal analysis, Investigation, Writing – review & editing. FS: Formal analysis, Investigation, Writing – review & editing. TO: Formal analysis, Investigation, Writing – review & editing. JP: Formal analysis, Investigation, Writing – review & editing. BP: Formal Analysis, Investigation, Writing – review & editing. IL: Investigation, Validation, Writing – review & editing. FS: Conceptualization, Funding acquisition, Supervision, Writing – review & editing. AQ: Formal analysis, Investigation, Writing – review & editing. FT: Formal analysis, Investigation, Writing – review & editing. SC: Formal analysis, Investigation, Writing – review & editing. NR: Formal analysis, Investigation, Writing – review & editing. JN: Formal analysis, Investigation, Writing – review & editing. SP: Formal analysis, Investigation, Writing – review & editing. LR: Formal analysis, Investigation, Writing – review & editing. LJ: Formal analysis, Investigation, Writing – review & editing. CB: Formal analysis, Investigation, Writing – review & editing. KS: Conceptualization, Investigation, Supervision, Writing – review & editing. LS: Conceptualization, Supervision, Writing – review & editing. TC: Conceptualization, Funding acquisition, Investigation, Supervision, Writing – review & editing.

## References

[1] Bray F Ferlay J Soerjomataram I Siegel RL Torre LA Jemal A. Global cancer statistics 2018: GLOBOCAN estimates of incidence and mortality worldwide for 36 cancers in 185 countries. CA Cancer J Clin (2018) 68(6):394–424. doi: 10.3322/caac.21492 30207593

[2] (ASCO) AS of CO. Bladder Cancer: Introduction (2020). Available at: https://www.cancer.net/cancer-types/bladder-cancer/introduction.

[3] Kamat AM Flaig TW Grossman HB Konety B Lamm D O’Donnell MA. Expert consensus document: Consensus statement on best practice management regarding the use of intravesical immunotherapy with BCG for bladder cancer. Nat Rev Urol (2015) 12:225–35. doi: 10.1038/nrurol.2015.58 25800393

[4] Kamat AM Hahn NM Efstathiou JA Lerner SP Malmström PU Choi W. Bladder cancer. Lancet (2016) 388(10061):2796–810. doi: 10.1016/S0140-6736(16)30512-8 27345655

[5] Barabas K Milner R Lurie D Adin C. Cisplatin: A review of toxicities and therapeutic applications. Veterinary Comp Oncol (2008) 6:1–18. doi: 10.1111/j.1476-5829.2007.00142.x 19178659

[6] Hoff PM Saad ED Costa F Coutinho AK Caponero R Prolla G. Literature review and practical aspects on the management of oxaliplatin-associated toxicity. Clin Colorectal Cancer (2012) 11:93–100. doi: 10.1016/j.clcc.2011.10.004 22154408

[7] Sievert KD Amend B Nagele U Schilling D Bedke J Horstmann M. Economic aspects of bladder cancer: What are the benefits and costs? World J Urol (2009) 27(3):295–300. doi: 10.1007/s00345-009-0395-z 19271220 PMC2694315

[8] Kobayashi T Owczarek TB McKiernan JM Abate-Shen C. Modelling bladder cancer in mice: Opportunities and challenges. Nat Rev Cancer (2015) 15:42–54. doi: 10.1038/nrc3858 25533675 PMC4386904

[9] Ding J Xu D Pan C Ye M Kang J Bai Q. Current animal models of bladder cancer: Awareness of translatability (Review). Exp Ther Med (2014) 8(3):691–9. doi: 10.3892/etm.2014.1837 PMC4113637 25120584

[10] Rangarajan A Weinberg RA. Comparative biology of mouse versus human cells: Modelling human cancer in mice. Nat Rev Cancer (2003) 3:952–9. doi: 10.1038/nrc1235 14737125

[11] Kendall SDS Linardic CM Adam SJ Counter CM. A network of genetic events sufficient to convert normal human cells to a tumorigenic state. Cancer Res (2005) 65(21):9824–8. doi: 10.1158/0008-5472.CAN-05-1543 16267004

[12] Rangarajan A Hong SJ Gifford A Weinberg RA. Species- and cell type-specific requirements for cellular transformation. Cancer Cell (2004) 6(2):171–83. doi: 10.1016/j.ccr.2004.07.009 15324700

[13] Sommer BC Dhawan D Ratliff TL Knapp DW. Naturally-occurring canine invasive urothelial carcinoma: A model for emerging therapies. Bladder Cancer (2018) 4:149–59. doi: 10.3233/BLC-170145 PMC5929349 29732386

[14] Norris AM Laing EJ Valli VEO Withrow SJ Macy DW Ogilvie GK. Canine bladder and urethral tumors: A retrospective study of 115 cases (1980–1985). J Vet Intern Med (1992) 6(3):145–53. doi: 10.1111/j.1939-1676.1992.tb00330.x 1619591

[15] Prather RS. Pig genomics for biomedicine. Nat Biotechnol (2013) 31(2):122–4. doi: 10.1038/nbt.2490 23392511

[16] Segatto NV Remião MH Schachtschneider KM Seixas1 FK Schook LB Collares T. The Oncopig cancer model as a complementary tool for phenotypic drug discovery. Front Pharmacol (2017) 8(894):1–8. doi: 10.3389/fphar.2017.00894 29259556 PMC5723300

[17] Xu C Wu S Schook LB Schachtschneider KM. Translating human cancer sequences into personalized porcine cancer models. Front Oncol (2019) 9. doi: 10.3389/fonc.2019.00105 PMC6401626 30873383

[18] Meurens F Summerfield A Nauwynck H Saif L Gerdts V. The pig: a model for human infectious diseases. Trends Microbiol (2012) 20(1):50–7. doi: 10.1016/j.tim.2011.11.002 PMC7173122 22153753

[19] Vandamme T. Use of rodents as models of human diseases. J Pharm Bioallied Sci (2014) 6(1):2–9. doi: 10.4103/0975-7406.124301 24459397 PMC3895289

[20] Groenen MA Archibald AL Uenishi H Tuggle CK Takeuchi Y Rothschild MF. Analyses of pig genomes provide insight into porcine demography and evolution. Nature (2012) 491(7424):393–8. doi: 10.1038/nature11622 PMC3566564 23151582

[21] Schachtschneider KM Madsen O Park C Rund LA Groenen MA Schook LB. Adult porcine genome-wide DNA methylation patterns support pigs as a biomedical model. BMC Genomics (2015) 16(743):1–18. doi: 10.1186/s12864-015-1938-x 26438392 PMC4594891

[22] Schachtschneider KM Schwind RM Newson J Kinachtchouk N Rizko M Mendoza-Elias N. The oncopig cancer model: an innovative large animal translational oncology platform. Front Oncol (2017) 7:190. doi: 10.3389/fonc.2017.00190 28879168 PMC5572387

[23] Schook LB Rund L Begnini KR Remiao MH Seixas FK Collares T. Emerging technologies to create inducible and genetically defined porcine cancer models. Front Genet (2016) 7:28. doi: 10.3389/fgene.2016.00028 26973698 PMC4770043

[24] Segatto NV Bender CB Seixas FK Schachtschneider K Schook L Robertson N. Perspective: humanized pig models of bladder cancer. Front Mol Biosci (2021) 8. doi: 10.3389/fmolb.2021.681044 PMC8165235 34079821

[25] Schook LB Collares TV Hu W Liang Y Rodrigues FM Rund LA. A genetic porcine model of cancer. PloS One (2015) 10(7):e0128864. doi: 10.1371/journal.pone.0128864 26132737 PMC4488487

[26] Schachtschneider KM Liu Y Makelainen S Madsen O Rund LA Groenen MAM. Oncopig soft-tissue sarcomas recapitulate key transcriptional features of human sarcomas. Sci Rep (2017) 7(1):1–12. doi: 10.1038/s41598-017-02912-9 28572589 PMC5453942

[27] Principe DR Overgaard NH Park AJ Diaz AM Torres C McKinney R. KRASG12D and TP53R167H cooperate to induce pancreatic ductal adenocarcinoma in sus scrofa pigs. Sci Rep (2018) 8(1):1–10. doi: 10.1038/s41598-018-30916-6 30135483 PMC6105629

[28] Schachtschneider KM Schwind RM Darfour-Oduro KA De AK Rund LA Singh K. A validated, transitional and translational porcine model of hepatocellular carcinoma. Oncotarget (2017) 8(38):63620–34. doi: 10.18632/oncotarget.18872 PMC5609948 28969016

[29] Chen J Wang L Tang Y Gong G Liu L Chen M. Maspin enhances cisplatin chemosensitivity in bladder cancer T24 and 5637 cells and correlates with prognosis of muscle-invasive bladder cancer patients receiving cisplatin based neoadjuvant chemotherapy. J Exp Clin Cancer Res (2016) 35(1):2. doi: 10.1186/s13046-015-0282-y 26733306 PMC4702361

[30] Lin JF Lin YC Tsai TF Chen HE Chou KY Hwang TIS. Cisplatin induces protective autophagy through activation of BECN1 in human bladder cancer cells. Drug Des Devel Ther (2017) 11:1517–33. doi: 10.2147/DDDT.S126464 PMC5439993 28553083

[31] Long X Xiong W Zeng X Qi L Cai Y Mo M. Cancer-associated fibroblasts promote cisplatin resistance in bladder cancer cells by increasing IGF-1/ERβ/Bcl-2 signalling. Cell Death Dis (2019) 10(5):375. doi: 10.1038/s41419-019-1581-6 31076571 PMC6510780

[32] Vallo S Michaelis M Rothweiler F Bartsch G Gust KM Limbart DM. Drug-resistant urothelial canc cell lines display diverse sensitivity profiles to potential second-line therapeutics. Transl Oncol (2015) 8(3):210–6. doi: 10.1016/j.tranon.2015.04.002 PMC4487788 26055179

[33] Brown JM Attardi LD. The role of apoptosis in cancer development and treatment response. Nat Rev Cancer (2005) 5(3):231–7. doi: 10.1038/nrc1560 15738985

[34] Konstantakou EG Voutsinas GE Karkoulis PK Aravantinos G Margaritis LH Stravopodis DJ. Human bladder cancer cells undergo cisplatin-induced apoptosis that is associated with p53-dependent and p53-independent responses. Int J Oncol (2009) 35(2):401–16. doi: 10.3892/ijo_00000353 19578756

[35] Ricci MS Zong W. Chemotherapeutic approaches for targeting cell death pathways. Oncologist (2006) 11(4):342–57. doi: 10.1634/theoncologist.11-4-342 PMC3132471 16614230

[36] Saalfrank A Janssen KP Ravon M Flisikowski K Eser S Steiger K. A porcine model of osteosarcoma. Oncogenesis (2016) 5(3):e210. doi: 10.1038/oncsis.2016.19 26974205 PMC4815050

[37] Toledo F Wahl GM. MDM2 and MDM4: p53 regulators as targets in anticancer therapy. Int J Biochem Cell Biol (2007) 39:1476–82. doi: 10.1016/j.biocel.2007.03.022 PMC2043116 17499002

[38] Shahbazi J Lock R Liu T. Tumor protein 53-induced nuclear protein 1 enhances p53 function and represses tumorigenesis. Front Genet (2013) 4. doi: 10.3389/fgene.2013.00080 PMC3652520 23717325

[39] Das S El-Deiry WS Somasundaram K. Regulation of the p53 homolog p73 by adenoviral oncogene E1A. J Biol Chem (2003) 278(20):18313–20. doi: 10.1074/jbc.M211704200 12639967

[40] Hershko T Chaussepied M Oren M Ginsberg D. Novel link between E2F and p53: Proapoptotic cofactors of p53 are transcriptionally upregulated by E2F. Cell Death Differ (2005) 12(4):377–83. doi: 10.1038/sj.cdd.4401575 15706352

[41] Salvador JM Brown-Clay JD Fornace AJ. Gadd45 in stress signaling, cell cycle control, and apoptosis. Adv Exp Med Biol (2013) 793:1–19. doi: 10.1007/978-1-4614-8289-5_1 24104470

[42] Cho HJ Park SM Hwang EM Baek KE Kim IK Nam IK. Gadd45b mediates Fas-induced apoptosis by enhancing the interaction between p38 and retinoblastoma tumor suppressor. J Biol Chem (2010) 285(33):25500–5. doi: 10.1074/jbc.M109.091413 PMC2919113 20558744

[43] Gazzaniga P Silvestri I Gradilone A Scarpa S Morrone S Gandini O. Gemcitabine-induced apoptosis in 5637 cell line: An *in-vitro* model for high-risk superficial bladder cancer. Anticancer Drugs (2007) 18(2):179–85. doi: 10.1097/CAD.0b013e328010ef47 17159604

[44] Elmore S. Apoptosis: A review of programmed cell death. Toxicol Pathol (2007) 35(4):495–516. doi: 10.1080/01926230701320337 17562483 PMC2117903

[45] Desouza M Gunning PW Stehn JR. The actin cytoskeleton as a sensor and mediator of apoptosis. Bioarchitecture (2012) 2(3):75–87. doi: 10.4161/bioa.20975 22880146 PMC3414384

[46] Povea-Cabello S Oropesa-Ávila M de la Cruz-Ojeda P Villanueva-Paz M de la Mata M Suárez-Rivero JM. Dynamic reorganization of the cytoskeleton during apoptosis: The two coffins hypothesis. Int J Mol Sci (2017) 18(11):2393. doi: 10.3390/ijms18112393 29137119 PMC5713361

[47] Chiarugi P Giannoni E. Anoikis: A necessary death program for anchorage-dependent cells. Biochem Pharmacol (2008) 76:1352–64. doi: 10.1016/j.bcp.2008.07.023 18708031

[48] Bai SW Herrera-Abreu MT Rohn JL Racine V Tajadura V Suryavanshi N. Identification and characterization of a set of conserved and new regulators of cytoskeletal organization, cell morphology and migration. BMC Biol (2011) 9:54. doi: 10.1186/1741-7007-9-54 21834987 PMC3201212

[49] Chardin P. Function and regulation of Rnd proteins. Nat Rev Mol Cell Biol (2006) 7:54–62. doi: 10.1038/nrm1788 16493413

[50] Muller PAJ Vousden KH. P53 mutations in cancer. Nat Cell Biol (2013) 15(1):28. doi: 10.1038/ncb2641 23263379

[51] Pylayeva-Gupta Y Grabocka E Bar-Sagi D. RAS oncogenes: Weaving a tumorigenic web. Nat Rev Cancer (2011) 11(11):761–74. doi: 10.1038/nrc3106 PMC3632399 21993244

[52] Saiki Y Yoshino Y Fujimura H Manabe T Kudo Y Shimada M. DCK is frequently inactivated in acquired gemcitabine-resistant human cancer cells. Biochem Biophys Res Commun (2012) 421(1):98–104. doi: 10.1016/j.bbrc.2012.03.122 22490663

[53] Sabini E Ort S Monnerjahn C Konrad M Lavie A. Structure of human dCK suggests strategies to improve anticancer and antiviral therapy. Nat Struct Biol (2003) 10(7):513–9. doi: 10.1038/nsb942 12808445

[54] Leisewitz A Zimmerma n E Huang M Jones S Yang J Graves L. Regulation of ENT1 expression and ENT1-dependent nucleoside transport by c-Jun N-terminal kinase. Biochem Biophys Res Commun (2011) 404(1):370–5. doi: 10.1016/j.bbrc.2010.11.125 PMC3018549 21145879

[55] Wright N Lee S. Structures of human ENT1 in complex with adenosine reuptake inhibitors. Nat Struct Mol Biol (2019) 26(7):599–606. doi: 10.1038/s41594-019-0245-7 31235912 PMC6705415

