# Supplementary material for: Oncopig bladder cancer cells recapitulate human bladder cancer treatment responses in vitro
Source: Front Oncol. 2024 Feb 26;14:1323422. doi: 10.3389/fonc.2024.1323422 (PMC10926022; doi:10.3389/fonc.2024.1323422)
Supplement: Supplementary file 1 [file DataSheet_1.pdf]

Supplemental Figures

Supplemental Figure 1. Oncopig BCCL characterization. (A) RT-PCR results demonstrating TP53^{R167H} and KRAS^{G12D} transgene expression in AdCre treated Oncopig bladder carcinoma cell lines (BCCLs) but not in the untreated Oncopig bladder control line (BCL). (B) Immunocytochemistry demonstrating positive Uroplakin II staining, a marker for urothelial carcinoma, in Oncopig BCCLs. Green represents Uroplakin-II staining and blue represents DAPI staining (cell nucleus).

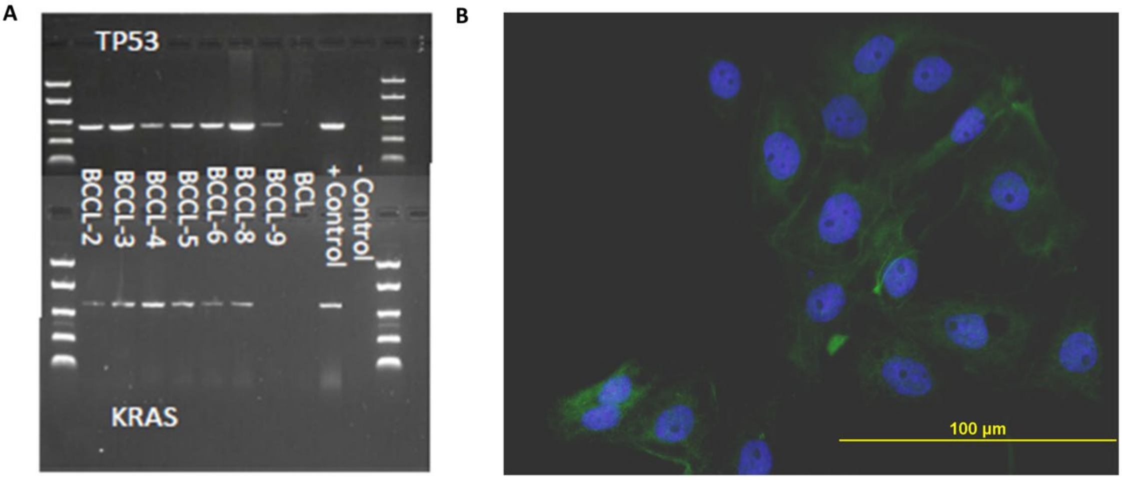

Supplemental Figure 2. Human and Oncopig cell line sensitivity to chemotherapeutics. Human bladder cancer cell lines (5637 and T24) and Oncopig BCCLs treated with commercial chemotherapeutics cisplatin, gemcitabine, or doxorubicin. (A) Confocal microscopy images of cells treated for 48h subjected to Live/Dead assay. Green represents calcein staining (live cells/emission ~ 515 nm) and red represents EthD-1 staining (dead cells/emission ~ 635nm). Magnification: 200x. (B) Flow cytometry analysis of total viable cells/mL of cells treated for 48h and subjected to ViaCount® assay, which distinguishes viable and nonviable cells based on differential permeability of two DNA binding dyes.

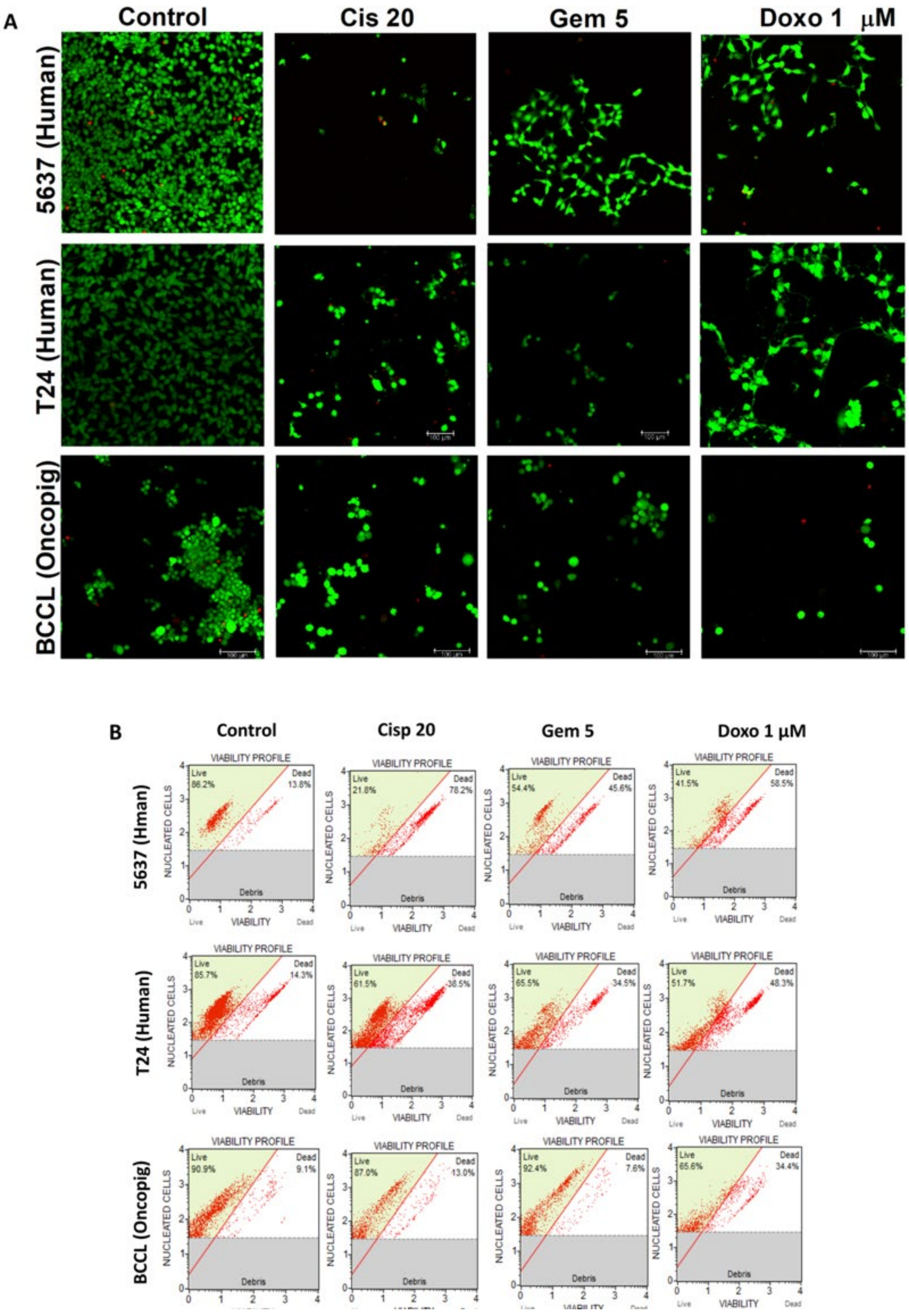

Supplemental Figure 3. Prediction of protein-ligand interactions assessed by docking. 3D (A , C and E) and 2D (B , D and F) representations of gemcitabine with human CYP450 (A and B), porcine CYP450 (C and D) and murine CYP450 (E and F).

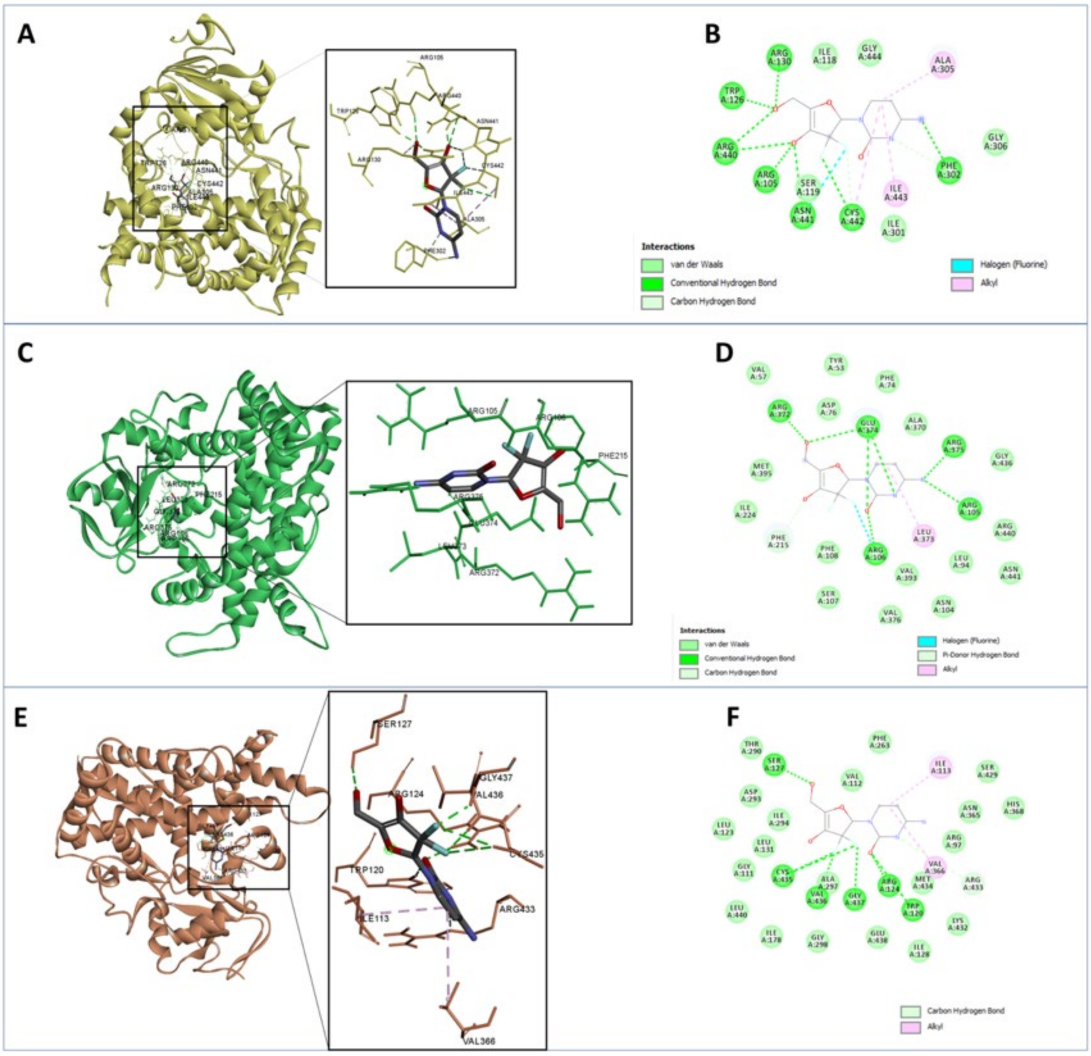

Supplemental Figure 4. Prediction of protein-ligand interactions assessed by docking. 3D (A , C and E) and 2D (B , D and F) representations of Doxorubicin with human CYP450 (A and B), porcine CYP450 (C and D) and murine CYP450 (E and F).

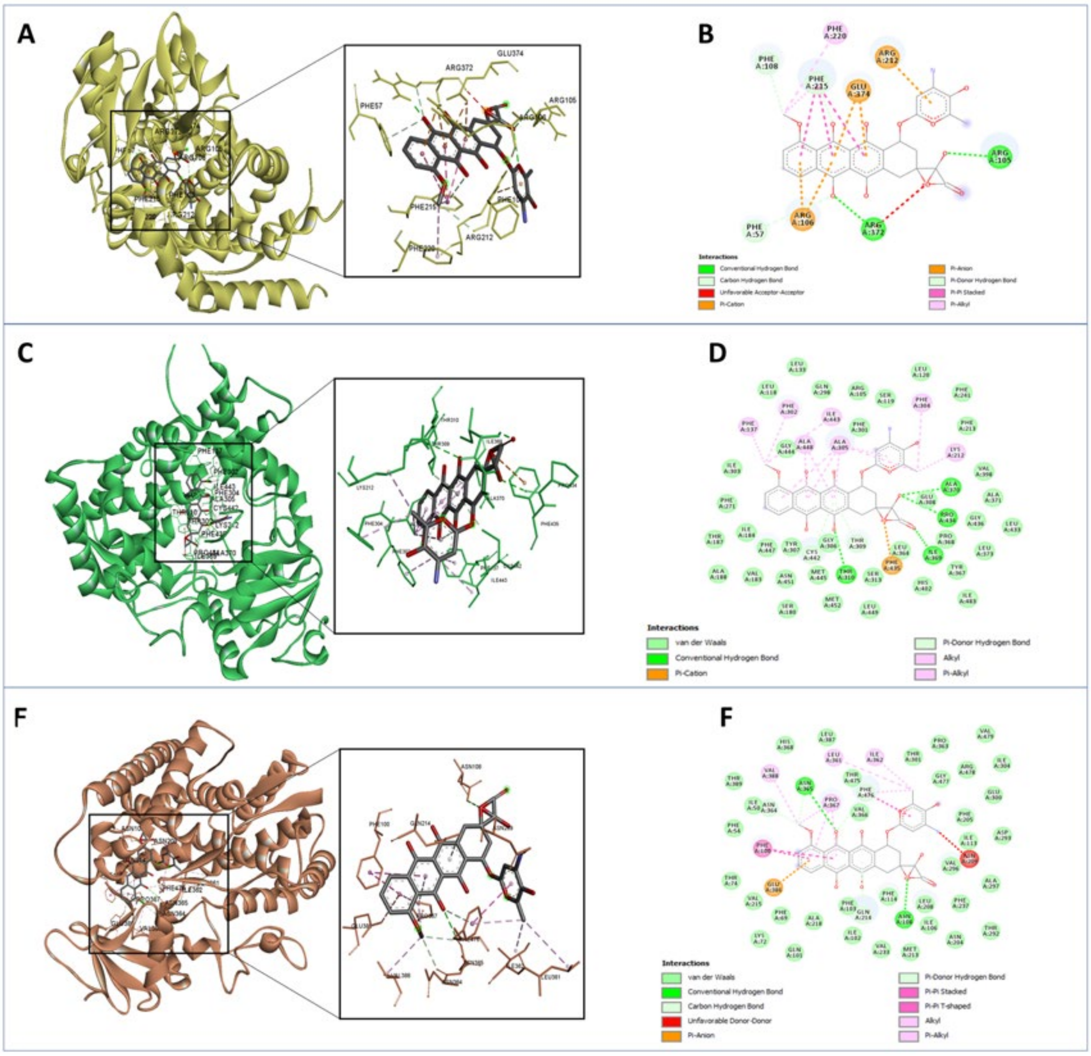

Supplemental Figure 5. Prediction of protein-ligand interactions assessed by docking. 3D representations (A , C and E) and binding residues (B , D and F) of Cisplatin with human CYP450 (A and B), porcine CYP450 (C and D) and murine CYP450 (E and F).

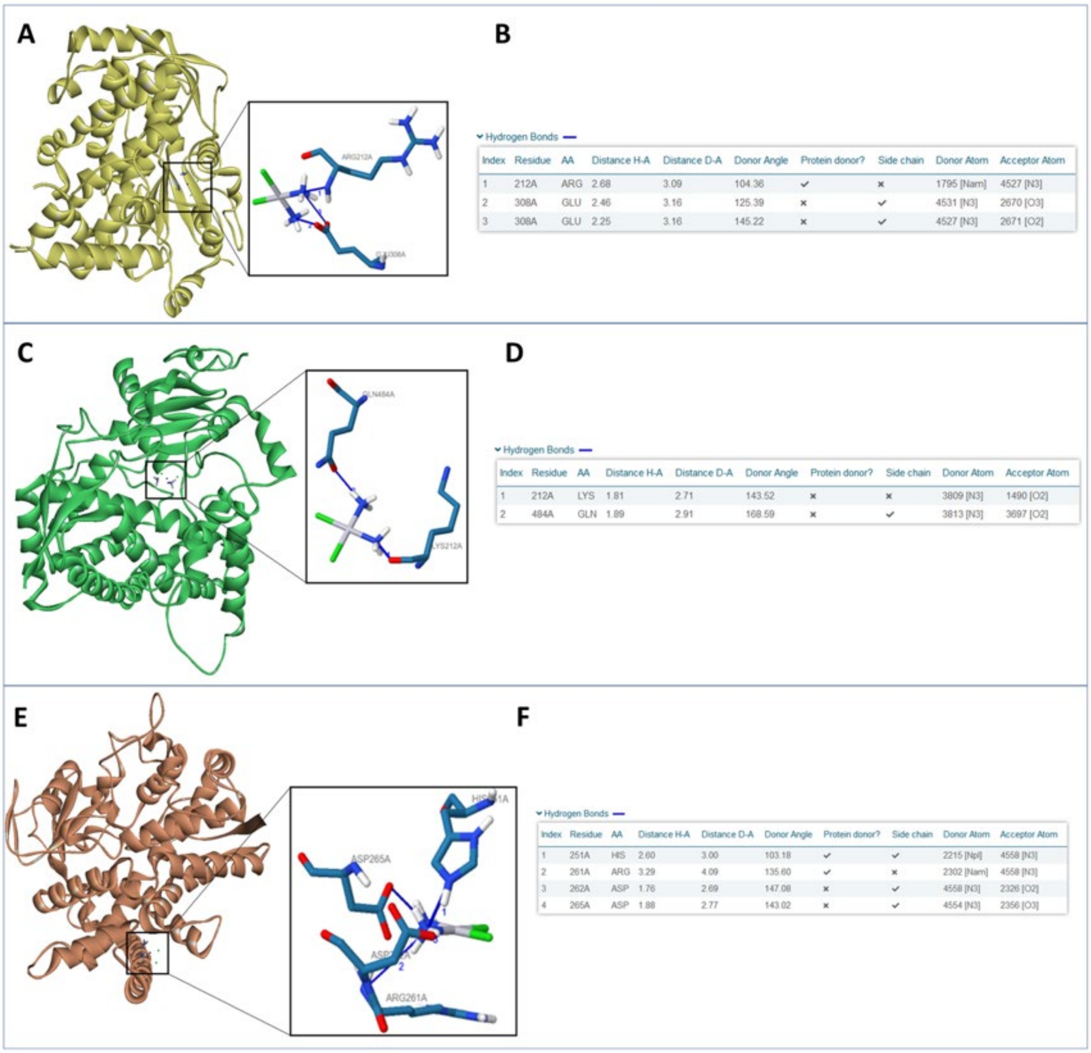

B — Hydrogen Bonds

Index	Residue	AA	Distance H-A	Distance D-A	Donor Angle	Protein donor?	Side chain	Donor Atom	Acceptor Atom
1	212A	ARG	2.68	3.09	104.36	✔	✖	1795 [Nam]	4527 [N3]
2	308A	GLU	2.46	3.16	125.39	✖	✔	4531 [N3]	2670 [O3]
3	308A	GLU	2.25	3.16	145.22	✖	✔	4527 [N3]	2671 [O2]

D — Hydrogen Bonds

Index	Residue	AA	Distance H-A	Distance D-A	Donor Angle	Protein donor?	Side chain	Donor Atom	Acceptor Atom
1	212A	LYS	1.81	2.71	143.52	✖	✖	3809 [N3]	1490 [O2]
2	484A	GLN	1.89	2.91	168.59	✖	✔	3813 [N3]	3697 [O2]

F — Hydrogen Bonds

Index	Residue	AA	Distance H-A	Distance D-A	Donor Angle	Protein donor?	Side chain	Donor Atom	Acceptor Atom
1	251A	HIS	2.60	3.00	103.18	✔	✔	2215 [Npl]	4558 [N3]
2	261A	ARG	3.29	4.09	135.60	✔	✖	2302 [Nam]	4558 [N3]
3	262A	ASP	1.76	2.69	147.08	✖	✔	4558 [N3]	2326 [O2]
4	265A	ASP	1.88	2.77	143.02	✖	✔	4554 [N3]	2356 [O3]

Supplemental Figure 6. Prediction of protein-ligand interactions assessed by docking. 3D (A , C and E) and 2D (B , D and F) representations of gemcitabine with human DCK (A and B), porcine DCK (C and D) and murine DCK (E and F).

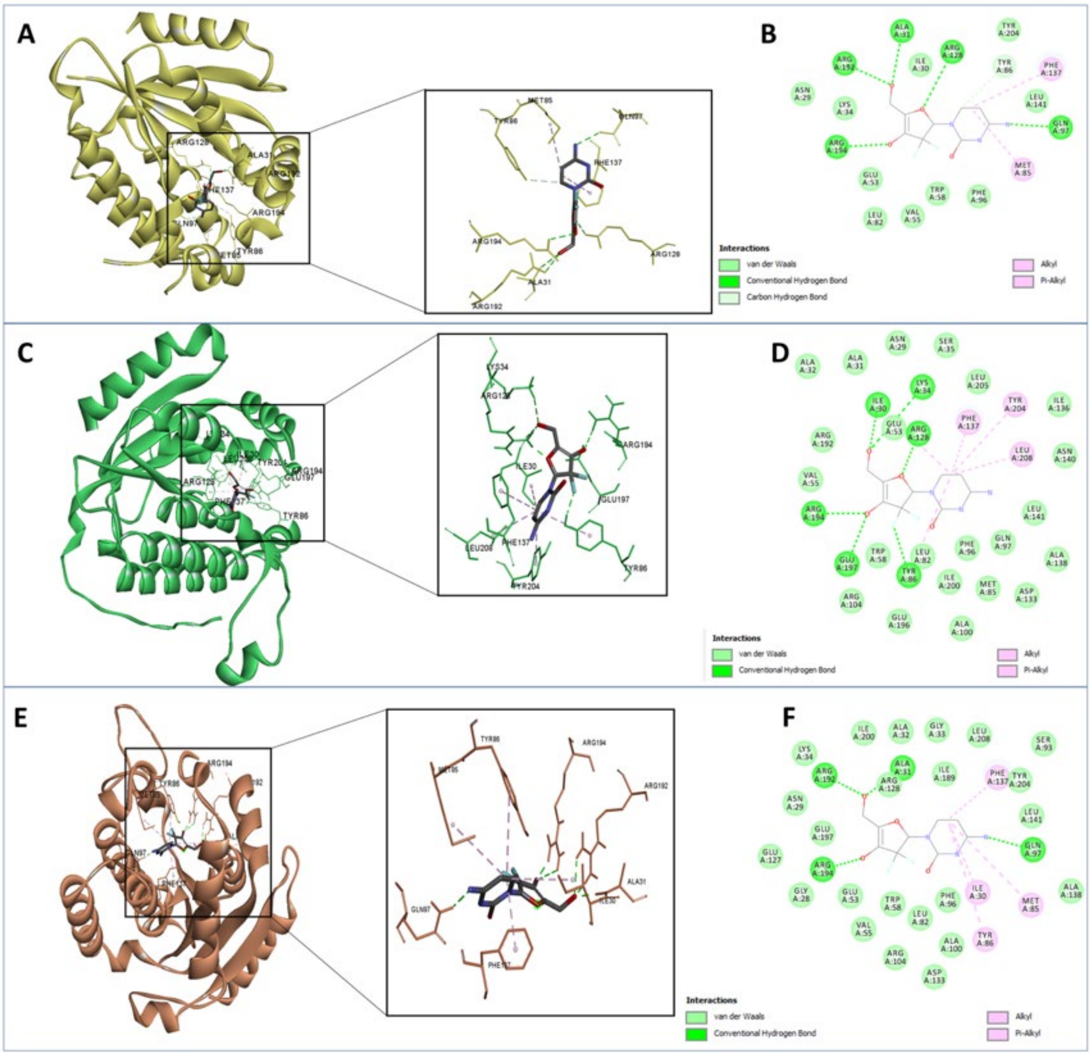

Supplemental Figure 7. Prediction of protein-ligand interactions assessed by docking. 3D (A , C and E) and 2D (B , D and F) representations of Gemcitabine with human ENT1 (A and B), porcine ENT1 (C and D) and murine ENT1 (E and F).

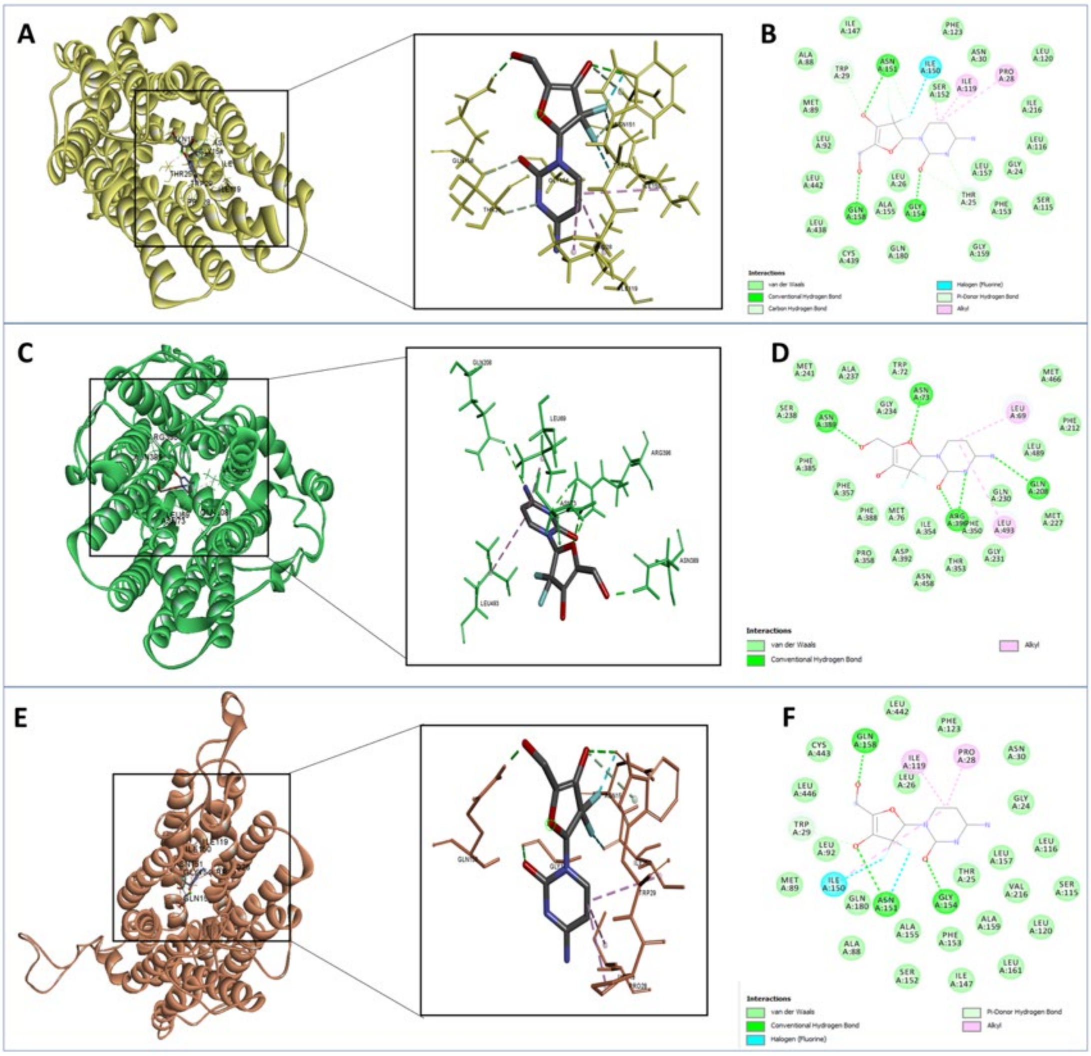

Supplemental Figure 8. Prediction of protein-ligand interactions assessed by docking. 3D (A , C and E) and 2D (B , D and F) representations of Gemcitabine with human CMPK1 (A and B), porcine CMPK1 (C and D) and murine CMPK1 (E and F).

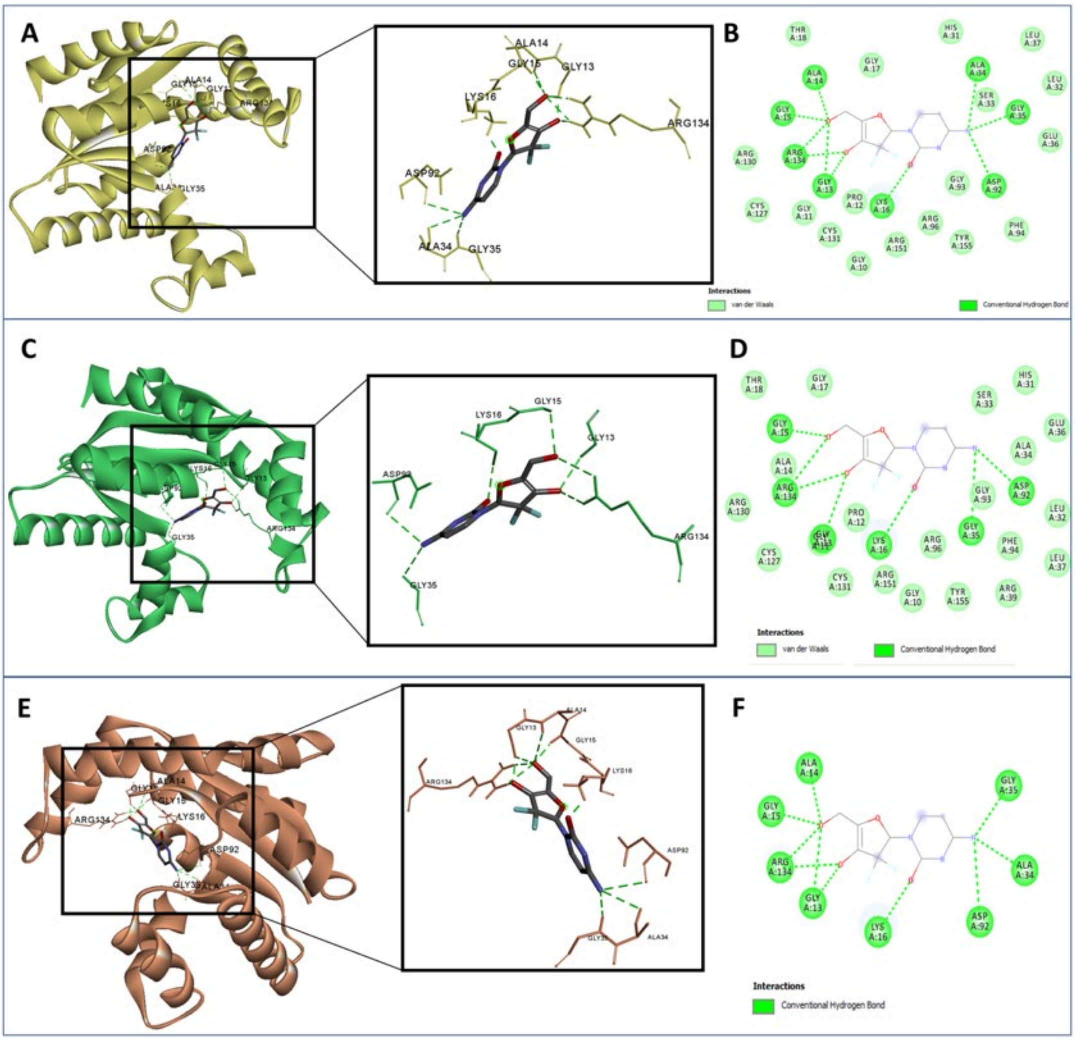

Supplemental Figure 9. Prediction of protein-ligand interactions assessed by docking. 3D (A , C and E) and 2D (B , D and F) representations of Doxorubicin with human AKR1A1 (A and B), porcine AKR1A1 (C and D) and murine AKR1A1 (E and F).

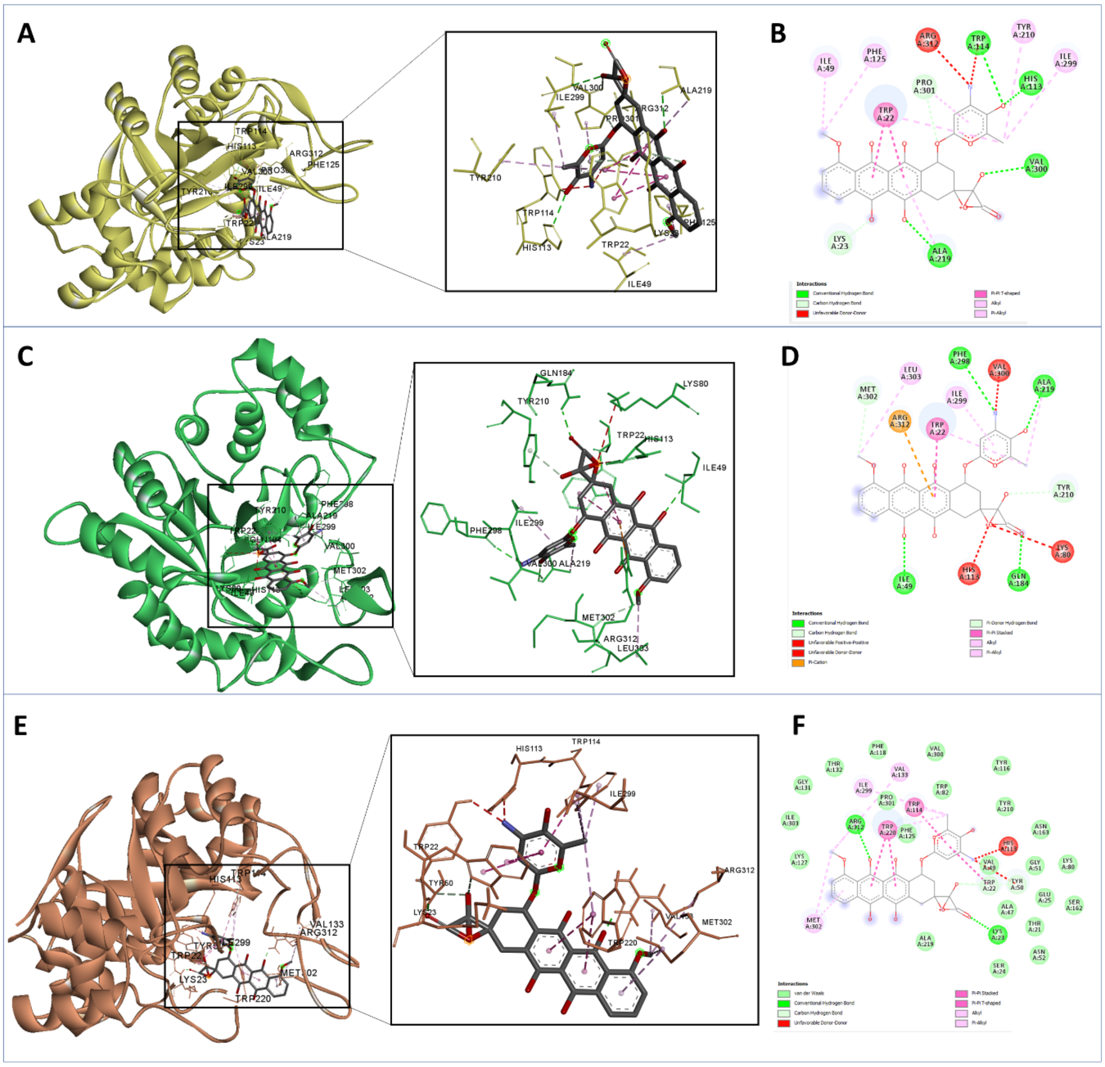

Supplemental Figure 10. Prediction of protein-ligand interactions assessed by docking. 3D (A , C and E) and 2D (B , D and F) representations of Doxorubicin with human NQO1 (A and B), porcine NQO1 (C and D) and murine NQO1 (E and F).

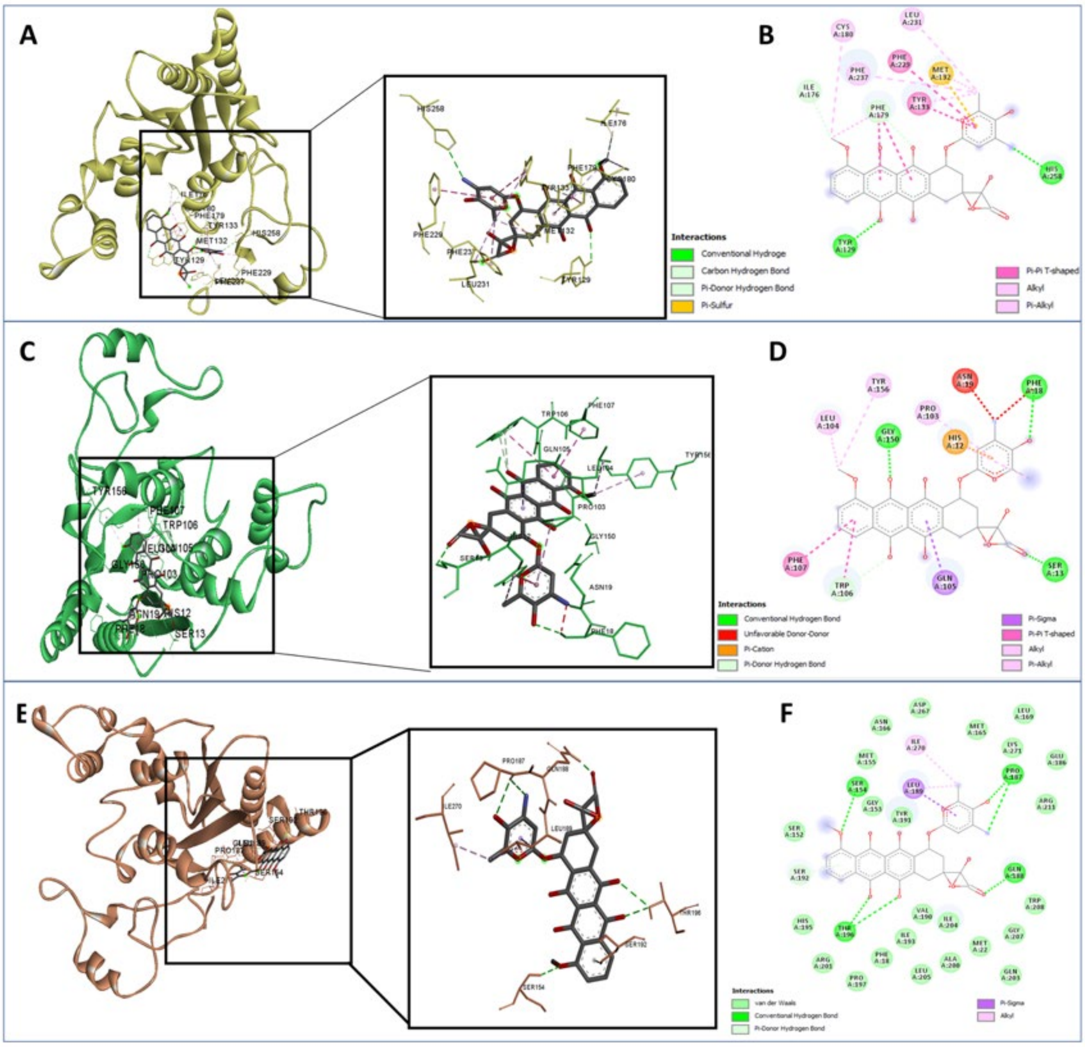

Supplemental Figure 11. Prediction of protein-ligand interactions assessed by docking. 3D representations (A , C and E) and binding residues (B , D and F) of Cisplatin with human PI3K (A and B), porcine PI3K (C and D) and murine PI3K (E and F).

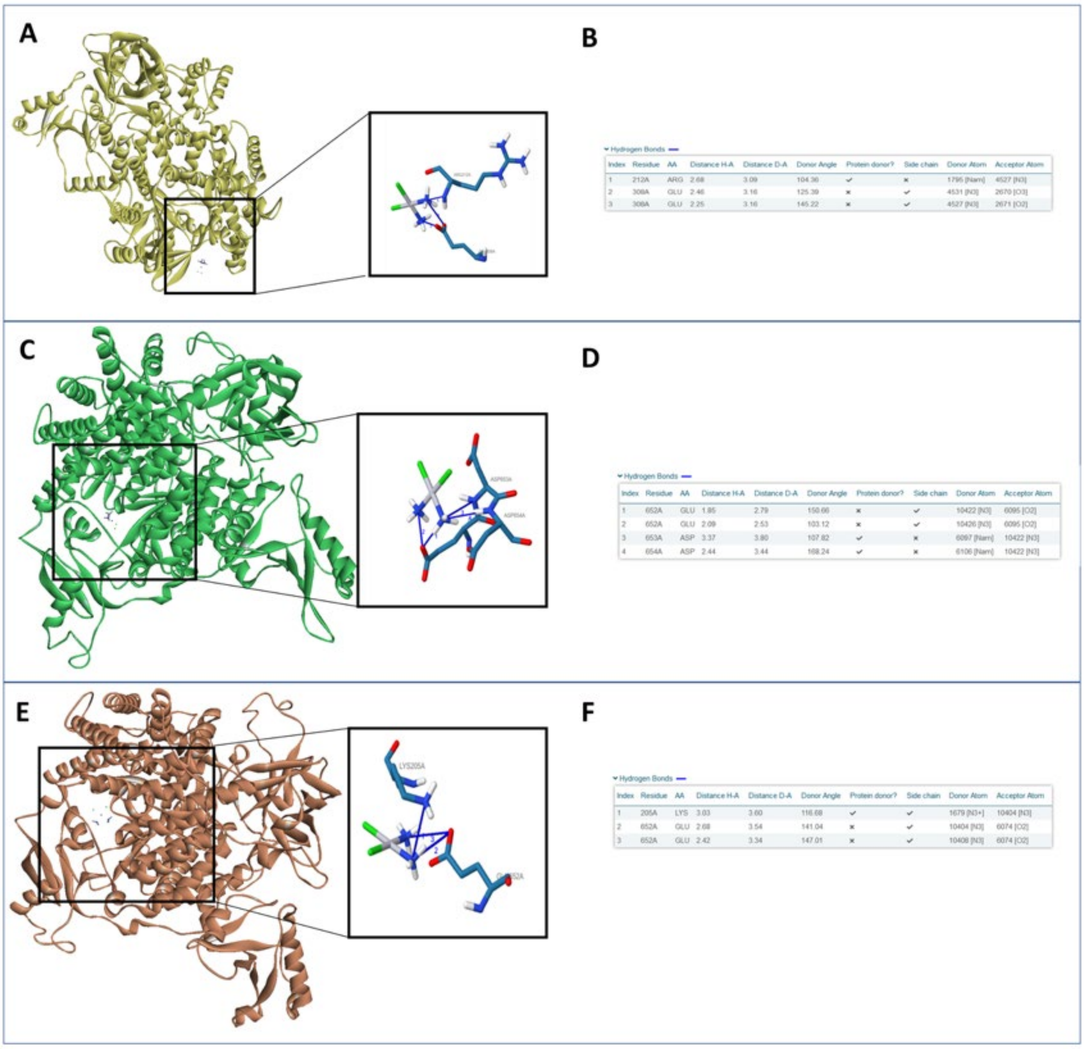

B — Hydrogen Bonds

Index	Residue	AA	Distance H-A	Distance D-A	Donor Angle	Protein donor?	Side chain	Donor Atom	Acceptor Atom
1	212A	ARG	2.68	3.09	104.36	✔	✖	1795 [Nam]	4527 [N3]
2	308A	GLU	2.46	3.16	125.39	✖	✔	4531 [N3]	2670 [O3]
3	308A	GLU	2.25	3.16	145.22	✖	✔	4527 [N3]	2671 [O2]

D — Hydrogen Bonds

Index	Residue	AA	Distance H-A	Distance D-A	Donor Angle	Protein donor?	Side chain	Donor Atom	Acceptor Atom
1	652A	GLU	1.85	2.79	150.66	✖	✔	10422 [N3]	6095 [O2]
2	652A	GLU	2.09	2.53	103.12	✖	✔	10426 [N3]	6095 [O2]
3	653A	ASP	3.37	3.80	107.82	✔	✖	6097 [Nam]	10422 [N3]
4	654A	ASP	2.44	3.44	168.24	✔	✖	6106 [Nam]	10422 [N3]

F — Hydrogen Bonds

Index	Residue	AA	Distance H-A	Distance D-A	Donor Angle	Protein donor?	Side chain	Donor Atom	Acceptor Atom
1	205A	LYS	3.03	3.60	116.68	✔	✔	1679 [N3+]	10404 [N3]
2	652A	GLU	2.68	3.54	141.04	✖	✔	10404 [N3]	6074 [O2]
3	652A	GLU	2.42	3.34	147.01	✖	✔	10408 [N3]	6074 [O2]

Supplemental Tables

Supplemental Table 1. AA residues that interacted with chemotherapeutics.

Protein	Human protein AA residues	Swine protein AA residues	Mouse protein AA residues	Chemotherapy drug
CYP450	TRP126, ARG130, ALA305, PHE302, ILE443, CYS442, ASN441, SER119, ARG105, ARG440	ARG372, GLU374, ARG375, ARG105, LEU373, ARG106	SER127, ILE113, VAL366, TRP120, ARG124, GLY437, VAL436, CYS435	gemcitabine
CYP450	ARG106, ARG372, ARG105, ARG212, GLU374, PHE215, PHE220	PHE137, PHE302, ILE443, ALA448, ALA305, PHE304, LYS212, ALA370, PRO434, ILE369, PHE435, THR310	ASN209, ASN108, GLU386, PHE100, VAL388, ASN365, PRO367, LEU361, ILE362, PHE476	doxorubicin
CYP450	ARG212, GLU308	LYS212, GLN484	HIS251, ARG261, ASP262, ASP265	cisplatin
DCK	MET85, GLN97, PHE137, ARG128, ALA31, ARG192, ARG194	ILE30, LYS34, ARG128, PHE137, TYR204, LEU208, LEU82, TYR86, GLU197, ARG194	ARG194, ARG192, ALA31, PHE137, MET85, GLN97, ILE30, TYR86	gemcitabine
ENT1	ASN151, ILE150, SER152, ILE119, PRO28, GLY154, GLN158	GLN208, LEU69, ASN73, ASN389, ARG396, LEU493	GLY154, PRO28, ILE119, GLN158, ASN151, ILE150	gemcitabine
CMPK1	GLY35, ALA34, ALA14, GLY15, ARG134, GLY13, LYS16, ASP92	ASP92, GLY35, LYS16, GLY13, ARG134, GLY15	ALA14, GLY15, ARG134, GLY13, LYS16, ASP92, ALA34, GLY35	gemcitabine
AKR1A1	ARG312, TRP114, HIS113, VAL300, ALA219, TRP22, PHE125, ILE49	LYS80, GLN184, HIS113, ILE49, LEU303, MET302, ARG312, TRP22, ILE299, PHE298, VAL300, ALA219	ILE299, VAL133, TRP114, HIS113, TYR50, TRP22, LYS23, MET302, ARG312,	doxorubicin
NQO1	HIS258, TYR129, MET132, TYR133, PHE229, LEU231, PHE237, CYS180, ILE176, PHE179	LEU104, TYR156, PRO103, HIS12, ASN19, PHE18, SER13, GLN105, TRP106, PHE107	PRO187, GLN188, THR196, SER154, LEU189, ILE270	doxorubicin
PI3K	ARG212, GLU308	ASP654, ASP653, GLU652	LYS205, GLU852	cisplatin
